# Supplementary material for: Binding of Myomesin to Obscurin-Like-1 at the Muscle M-Band Provides a Strategy for Isoform-Specific Mechanical Protection
Source: Structure. 2017 Jan 3;25(1):107–20. doi: 10.1016/j.str.2016.11.015 (PMC5222588; doi:10.1016/j.str.2016.11.015)
Supplement: Document S1. Supplemental Experimental Procedures and Figures S1–S8 [file mmc1.pdf]

Structure, Volume *25*

Supplemental Information

Binding of Myomesin to Obscurin-Like-1 at the Muscle M-Band Provides a Strategy for Isoform-Specific Mechanical Protection

Stefano Pernigo, Atsushi Fukuzawa, Amy E.M. Beedle, Mark Holt, Adam Round, Alessandro Pandini, Sergi Garcia-Manyes, Mathias Gautel, and Roberto A. Steiner

Supplemental information for

Binding of myomesin to obscurin-like-1 at the muscle M-band provides a strategy for isoform-specific mechanical protection

Stefano Pernigo[1], Atsushi Fukuzawa[1,2], Amy E. M. Beedle[1,3], Mark Holt[1,2], Adam Round[4], Alessandro Pandini[1,5], Sergi Garcia-Manyes[1,3,*], Mathias Gautel[1,2,*], Roberto A. Steiner[1,*]

[1]Randall Division of Cell and Molecular Biophysics, King's College London, London, SE1 1UL, UK

[2]Cardiovascular Division, King's College London BHF Centre of Research Excellence, London, SE1 1UL, UK

[3]Department of Physics, King’s College London, London, WC2R 2LS, UK

[4]European Molecular Biology Laboratory, Grenoble Outstation, 38042 Grenoble, France

[5]Department of Computer Science and Synthetic Biology Theme, Brunel University London, UB8 3PH, UK

Contact

Correspondence should be addressed to

Email: roberto.steiner@kcl.ac.uk

Email: mathias.gautel@kcl.ac.uk

Email: sergi.garcia-manyes@kcl.ac.uk

Lead author

Email: roberto.steiner@kcl.ac.uk

SI Materials and Methods

Cloning

The sequence for the third Ig domain of either human obscurin (residues 238-334, O3) or human obscurin-like 1 (residues 251-339, OL3) was subcloned between the EcoRI/SalI sites of the first expression cassette of an in-house generated pGEX-6P-2rbs vector (a kind gift of the Musacchio Lab, MPI, Dortmund). The pGEX-6P-2rbs vector is based on the pGEX-6P vector (GE Healthcare) but contains two expression cassettes separated by a ribosome binding site. Proteins expressed from the first cassette are C-terminal fusions to GST. The sequence for human myomesin encompassing the fourth and fifth Fn-III domains (residues 510-739, My4*L*My5) was cloned between the BglII/XhoI restriction sites of the second expression cassette. Constructs expressing a deletion version of My4*L*My5 in which the fifth Fn-III domain is removed (residues 510-635, My4*L*) were generated using the QuikChange kit (Stratagene) by replacing the triplet coding for residue I636 with a stop codon. Additionally, cDNA corresponding to myomesin residues 510-618 ($My4L_H$) was cloned in an in-house modified pET9 vector. Downstream the His_6-tag, this vector encodes for a tobacco etch virus (TEV) protease recognition sequence. All constructs were verified by sequencing.

Protein expression and purification

All vectors were independently transformed into the BL21 (DE3) *E. coli* strain to allow protein expression using standard methods. GST-obscurin(-like-1):myomesin complexes were purified by incubating the soluble fraction with Glutathione Superflow beads (Generon) previously equilibrated in lysis buffer for 3h at 4° C. After incubation the beads were extensively washed and resuspended in lysis buffer.

The GST tag was removed by O/N incubation of the beads suspension with PreScission protease (GE healthcare) at 4° C with gentle shaking. Untagged complexes were separated from the resin–bound protein material by centrifugation at 400g for 15 minutes. All complexes were further purified by ion-exchange chromatography (IEC) and extensively dialyzed at 4°C against 50 mM Tris/HCl, 15 mM NaCl, 1.0 mM DTT, pH 8.5 and 50 mM MES, 15 mM NaCl, 1.0 mM DTT, pH 6.0, respectively. The O3:My4*L*(My5): and OL3:My4*L*(My5) complexes were loaded onto ResourceQ and ResourceS columns (GE healthcare), respectively, and eluted using a NaCl linear gradient. Fractions containing the complexes were pooled, concentrated and further purified by size-exclusion chromatography (SEC) on a 16⁄60 HiLoad Superdex 75 column (GE Healthcare) equilibrated with 20 mM Hepes, 50 mM NaCl, 1 mM DTT, pH 7.5. His_6-My4L_H was purified by immobilised metal affinity purification (IMAC) followed by His_6-tag cleavage using TEV protease. Untagged My4L_H was separated from the uncleaved material by a second round of IMAC. Flow-through fractions containing My4α were pooled, concentrated and further purified by (SEC) on a 16⁄60 HiLoad Superdex 75 column (GE Healthcare) equilibrated with 20 mM HEPES, 150 mM NaCl, 1 mM DTT, pH 7.5.

Crystallisation of My4L_H

Crystallization of My4L_H was accomplished using the vapor-diffusion method by concentrating the protein at 20 mg/ml. Optimization of hits obtained using various commercial screens produced diffracting crystals belonging to space group $P2_1$ and $P6_5$ in 25% (w/v) PEG 3350, 0.1 M BIS/Tris pH 5.5 and 21% (w/v) PEG 2000 MME, 0.1 M Tris-HCl pH 8.8, 0.010 M $NiCl_2$, respectively.

X-ray data collection and structure determination

For the OL3:My4*L*My5 complex Matthews' coefficient calculations (Matthews, 1968) suggested a V_M of 2.87 Å^{3}Da^{-1} as the most probable value. This implied the presence of two OL3:My4*L*My5 heterodimers in the a.u. with a solvent content of 57.2%. Using the Fn-III domain of 2NZI (Mrosek et al., 2007) as template, molecular replacement (MR) attempts performed using the program *MOLREP* (Vagin and Teplyakov, 2010) identified two Fn-III domains related by a 2-fold symmetry axis along the z axis. Further attempts at locating two additional Fn-III domains by MR were unsuccessful. We then decided to turn our attention to the OL3 domain. Using a truncated poly-Ala version of the OL1 Ig domain (PDB code 2WP3 (Pernigo et al., 2010), a new MR search positioned two Ig domains obeying the same dyad symmetry of the previously positioned Fn-III domains. Model building was carried out manually using the program *COOT* (Emsley et al., 2010) and structure refinement was performed using the programs *REFMAC*5 (Murshudov et al., 2011) and *BUSTER* (Bricogne et al., 2011). Electron density maps calculated during the refinement process failed to reveal information on the missing Fn-III domains. The final deposited structure (PDB code, 5FM5) consists of a pair of OL3:My4*L* heterodimers assembled as a dimer in the crystal. The structure of My4L_H was solved in space groups $P6_5$ and $P2_1$ by MR starting from the relevant portion of 5FM5 as template. Final models have been deposited with the PDB with accession codes 5FM8 and 5FM4, respectively. A summary of refinement statistics is shown in (Table 1).

Cellular competition assays in NRCs and ratiometric analysis

Briefly, to see the competitive effect of overexpressed GFP-My4*L*My5 and their variants on endogenous obscurin, we imported 16-bit-per-channel RGB files into Mathematica 10 (Wolfram Research) and translated them to ensure correct alignment

of the myomesin and obscurin channels. Background subtraction was performed on these channels by subtracting a 50-pixel radius Gaussian filtered version of that channel. The arctangent of the obscurin and myomesin intensities was then calculated on a pixel-by-pixel basis to give a ratio image of obscurin to myomesin that was linearly scaled between 0.0 and 1.0. A value of 0.0 corresponded to no obscurin in the presence of myomesin, while a value of 1.0 corresponded to obscurin in the absence of myomesin. A value of 0.5 corresponded to equal intensities in both channels. The GFP channel was binarised to create a segmented image corresponding to GFP-positive and GFP-negative regions. The ratio image was multiplied by this mask and separately with an inverted version of this mask to give two ratio images that corresponded to the GFP-positive and GFP-negative regions of the image as two separate images. A histogram for each image was then determined and normalized to a total of 1 for each histogram. Plotting these against each other thus allowed comparison in shifts in ratio. A left shift (towards zero) indicated a reduction of obscurin in the GFP-positive ratio image. The difference between the two curves in this direction was calculated and the data presented represent one minus the total integrated difference between these two. Thus, a value of 1.0 represents total competition of endogenous obscurin by the GFP fusion, while a value of 0.0 represented absence of competition. Data in Figure 5B are reported normalized to the obscurin competition level of GFP-My4LMy5wt (0.504±0.092, mean±s.e.m., n=11).

Small angle X-ray scattering (SAXS)

All experiments were performed at 18 °C using a sample volume of 30 μl loaded into the flowing measurement cell. Individual frames were processed automatically and independently within the *EDNA* framework, yielding individual radially averaged curves of normalized intensity. Matched buffer measurements taken before and after

every sample were averaged and used for background subtraction. Merging of separate concentrations and further analysis steps were performed using a combination of tools from the *ATSAS* package (Petoukhov et al., 2012). Standard procedures were applied for the computation of the forward scattering $I(0)$, radius of gyration R_g, and maximum dimension D_{max}. The molecular mass of the solutes was evaluated by comparison with reference solutions of BSA. For *ab initio* envelope reconstruction a total of 40 models were calculated for each construct using *DAMMIF* (Franke and Svergun, 2009), and then averaged, aligned and compared using *DAMAVER* (Volkov and Svergun, 2003), the output of which was used in the final refinement to the scattering data with *DAMMIN* (Svergun, 1999). Initial rigid body modelling of the complex was done with *CORAL* (Petoukhov et al., 2012) and domain dynamics of the protein complexes was further explored by generating conformational ensembles using the *tCONCOORD* (Seeliger et al., 2007) method. A total of 32768 structures were generated for each complex with this method. Theoretical scattering and comparison to the experimental data was done with *PepsiSAXS*.

Single-molecule mechanical experiments by atomic force microscopy (AFM)

An N-terminal His_6 tag is also present to facilitate protein purification and two C-terminal Cys residues are available at the protein C-terminus to allow protein attachment to the atomic force microscope. The His_6-Ub-*L*-connector-OL3-Ub-CC polyproteins were purified using a combination of IMAC, IEC and SEC techniques. Constant velocity AFM experiments were conducted at room temperature using both a home-made set-up (Schlierf et al., 2004) and a commercial Luigs and Neumann force spectrometer (Popa et al., 2013). In all cases, the sample was prepared by depositing 1-10 μL of protein in PBS solution (at a concentration of 1-10 mg ml^{-1})

onto a freshly evaporated gold slide. Each cantilever (Si_3N_4 Bruker MLCT-AUHW) was individually calibrated using the equipartition theorem, giving rise to a typical spring constant of ~12 pN nm^{-1}.

Figure S1.

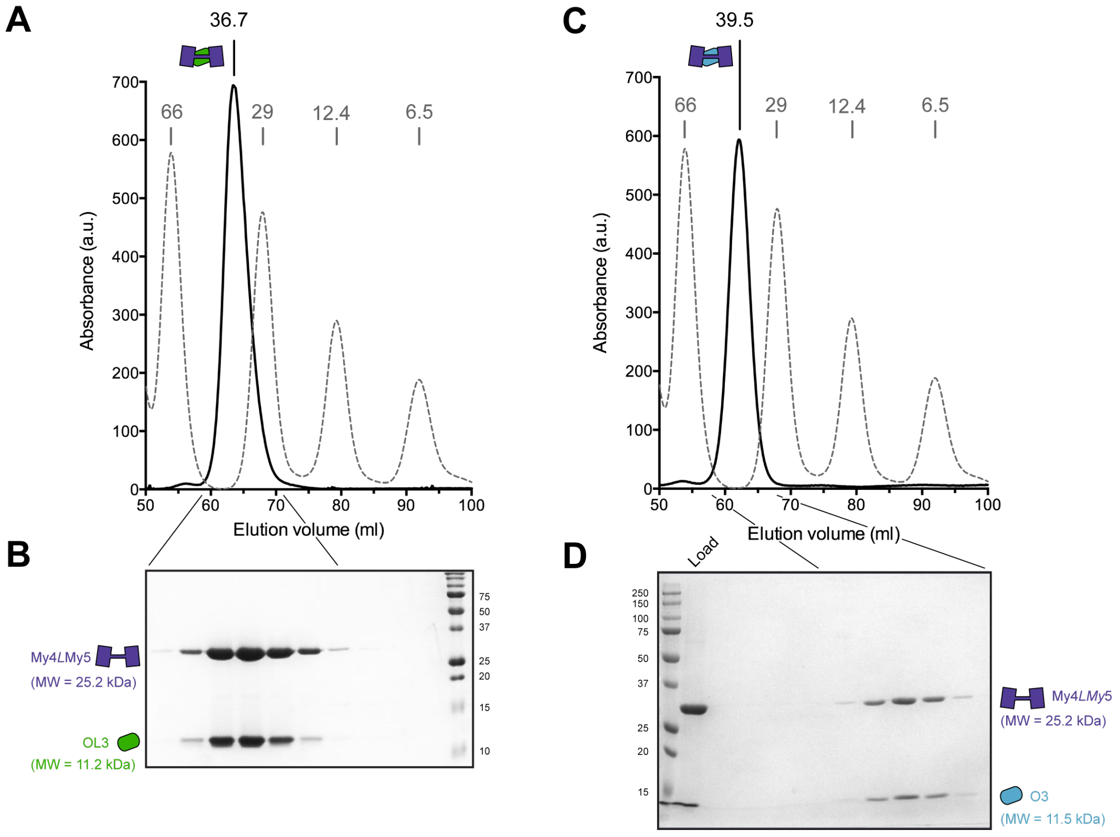

Figure S1 (related to Figure 1B, inset). OL3:My4*L*My5 and O3:My4*L*My5 complexes. (A,C) During purification OL3:My4*L*My5 (A) and O3:My4*L*My5 (C) (black continuous traces) elute as 1:1 stoichiometric complexes with an apparent molecular weight (MW) of 36.7 kDa and 39.5, respectively, from a Superdex 75 16/600 size-exclusion column. The calculated MW is 36.4 kDa and 36.8 kDa for OL3:My4*L*My5 and O3:My4*L*My5, respectively. The elution profile of calibration MW markers is shown with a broken grey line together with their MW in kDa; (B, D) Fractions of the OL3:My4*L*My5 (B) and O3:My4*L*My5 (D) peaks were analyzed by SDS–PAGE.

Figure S2.

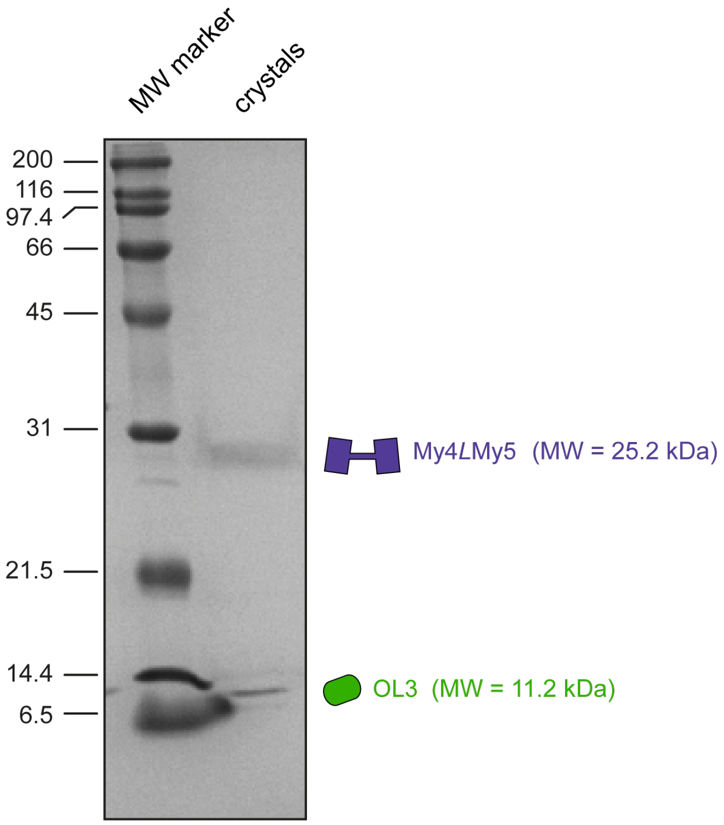

Figure S2. The OL3:My4*L*My5 complex is not proteolyzed in the crystal (related to Figure 1B, inset). OL3:My4*L*My5 crystals were extensively washed, dissolved in buffer, and analyzed by SDS-PAGE. Both My4*L*My5 and OL3 domains migrate according to their expected molecular weight (MW) indicating that proteolysis did not occur during the crystallization process. The lack of electron density for the My5 domain in crystallographic maps is likely due to flexibility of the hinge region connecting My4*L* to My5. The MW of protein markers is given on the left-hand.

Figure S3.

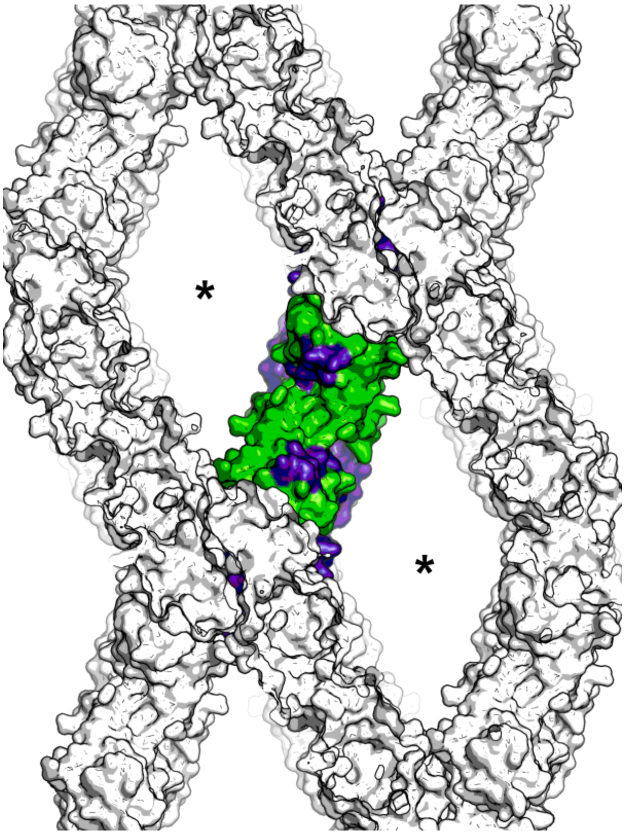

Figure S3. OL3:My4*L*My5 crystal packing (related to Figure 2A). A sliced view of the molecular arrangement in the crystal represented along the 2-fold $(OL3:My4L)_2$ axis. Large channels, highlighted by asterisks, can accommodate the My5 domain. A single $(OL3:My4L)_2$ complex is highlighted with My4*L* shown in slate blue and OL3 in green. Symmetry related molecules are represented in white.

Figure S4.

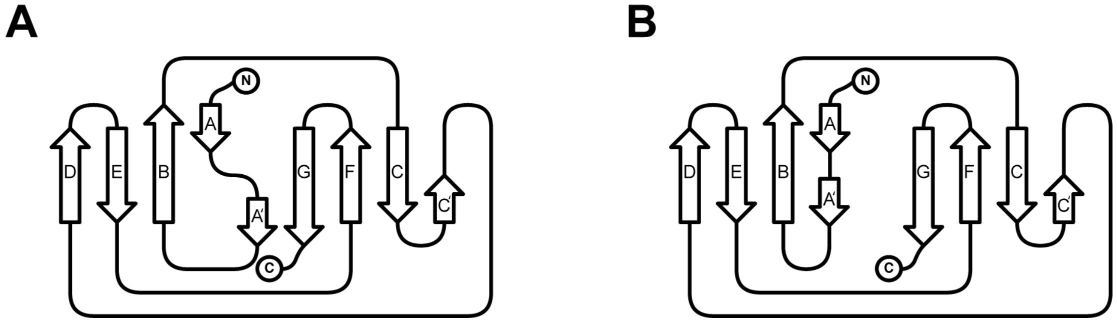

Figure S4. Intermediate-set (I-set) Ig topologies (related to Figure 3C, inset). Topology diagrams of (A) I-set and (B) I*-set Ig-fold domains. The I*-set exhibits a relocation of the A' strand (Pernigo et al., 2015). Strands are labelled according to the classical Ig-fold nomenclature (Harpaz and Chothia, 1994).

Figure S5.

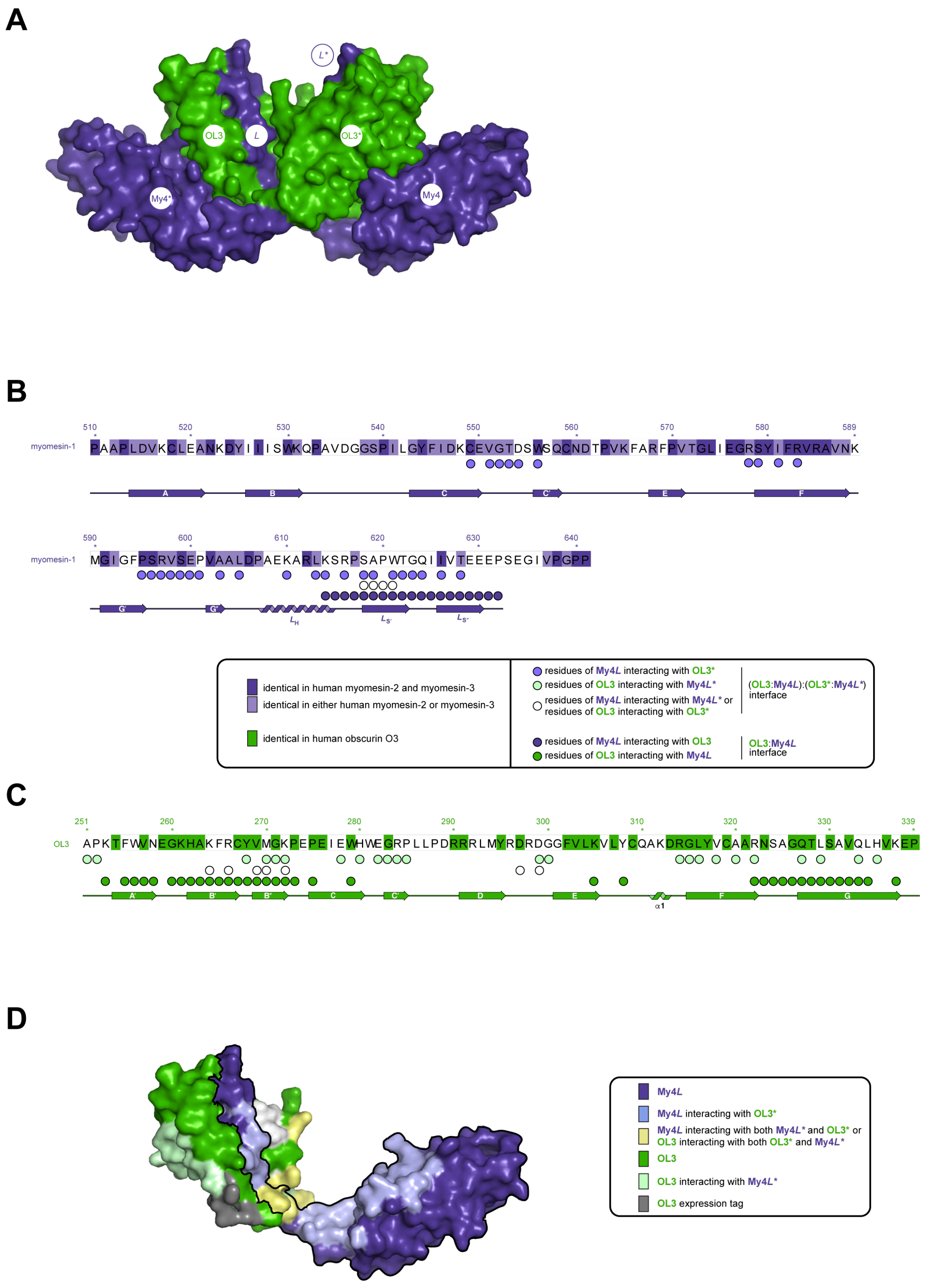
A
L*
OL3
L
OL3*
My4*
My4
B
myomesin-1
510 520 530 540 550 560 570 580 589
PAAPLDVKCLEANKDYIIISWKQPAVDGGSPILGYFIDKCEVGTDSWSQCNDTPVKFARFPVTGLIEGRSYIFRVRAVNK
A B C C' E F
590 600 610 620 630 640
MGIGFPSRVSEPVAALDPAEKARLKSRPSAPWTGQIIVTEEEPSEGIVPGPP
G G' L_H $L_{S'}$ $L_{S''}$
identical in human myomesin-2 and myomesin-3
identical in either human myomesin-2 or myomesin-3
identical in human obscurin O3
residues of My4L interacting with OL3*
residues of OL3 interacting with My4L*
residues of My4L interacting with My4L* or residues of OL3 interacting with OL3*
(OL3:My4L):(OL3*:My4L*) interface
residues of My4L interacting with OL3
residues of OL3 interacting with My4L
OL3:My4L interface
C
OL3
251 260 270 280 290 300 310 320 330 339
APKTFWVNEGKHAKFRCYVMGKPEPEIEWHWEGRPLLPDRRRLMYRDRDGGFVLKVLYCQAKDRGLYVCAARNSAGQTLSAVQLHVKEP
A' B' B'' C C' D E α1 F G
D
My4L
My4L interacting with OL3*
My4L interacting with both My4L* and OL3* or OL3 interacting with both OL3* and My4L*
OL3
OL3 interacting with My4L*
OL3 expression tag

Figure S5 (related to Figure 2A). (OL3:My4*L*)$_2$ homodimer. (A) Surface representation of the crystallographic (OL3:My4*L*)$_2$ homodimer. My4*L* and OL3 are in slate blue and green, respectively. An asterisk is used to identify domains belonging to the second OL3:My4*L* complex; (B-C) Sequence and secondary structure of My4*L* (B) and OL3 (C). Amino acids are color-coded according to sequence identity as shown by the conservation bar. Colored circles identify residues of one domain contacting the other as indicated in the inset; (D) Homodimerization contact map. Light blue, light green, and yellow patches on a single OL3:My4*L* complex highlight residues interacting with My4*L**, OL3*, or both domains, respectively. The outline of My4*L* is shown as a thick black line for clarity.

Figure S6.

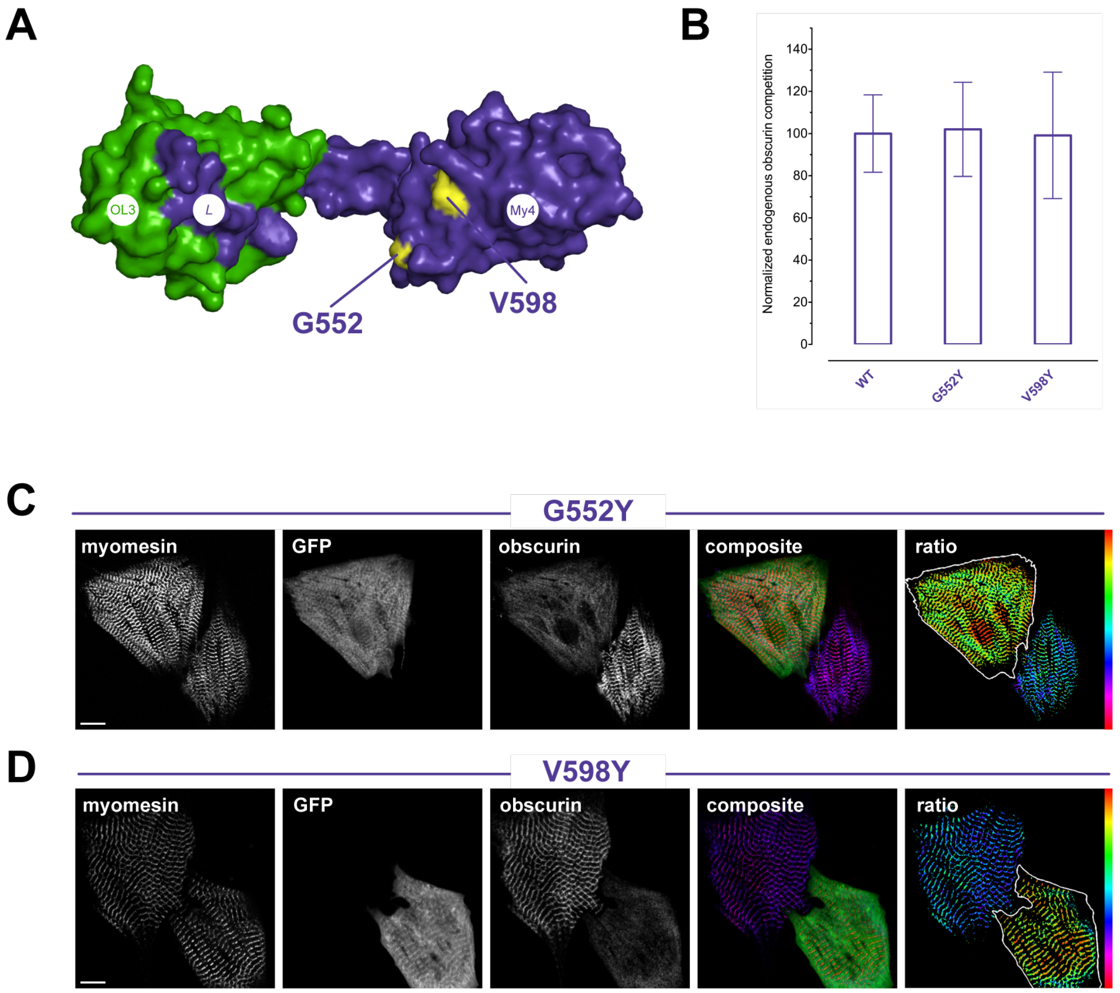
A
OL3
L
My4
G552
V598
B
Normalized endogenous obscurin competition
140
120
100
80
60
40
20
0
WT
G552Y
V598Y
C
G552Y
myomesin
GFP
obscurin
composite
ratio
D
V598Y
myomesin
GFP
obscurin
composite
ratio

Figure S6 (related to Figure 4). Controls for the competition assay in NRCs. (A) Surface representation of the OL3:My4*L* complex. Residues G552 and V598 that are not involved in establishing the OL3:My4*L* interface are highlighted in yellow. (B) Quantification of endogenous obscurin displaced in NRCs expressing GFP-fused wild-type My4*L*My5 (*n*=11) and its G552Y (n=10) and V598Y (*n*=10) variants. As expected, these variants compete endogenous obscurin like the wild-type counterpart as their amino acid replacement does not interfere with OL3 binding; (C-D) Example of the competitive effect of overexpressed GFP-fused My4*L*My5 G552Y (C) and V598Y (D). The separate channels for endogenous myomesin, GFP, endogenous obscurin, as well as the combined and ratiometric images with overlaid GFP mask for the outline of the transfected cell are shown. The false-color scale range indicator shows an increased obscurin/myomesin ratio. The scale-bar is 10 μm.

Figure S7.

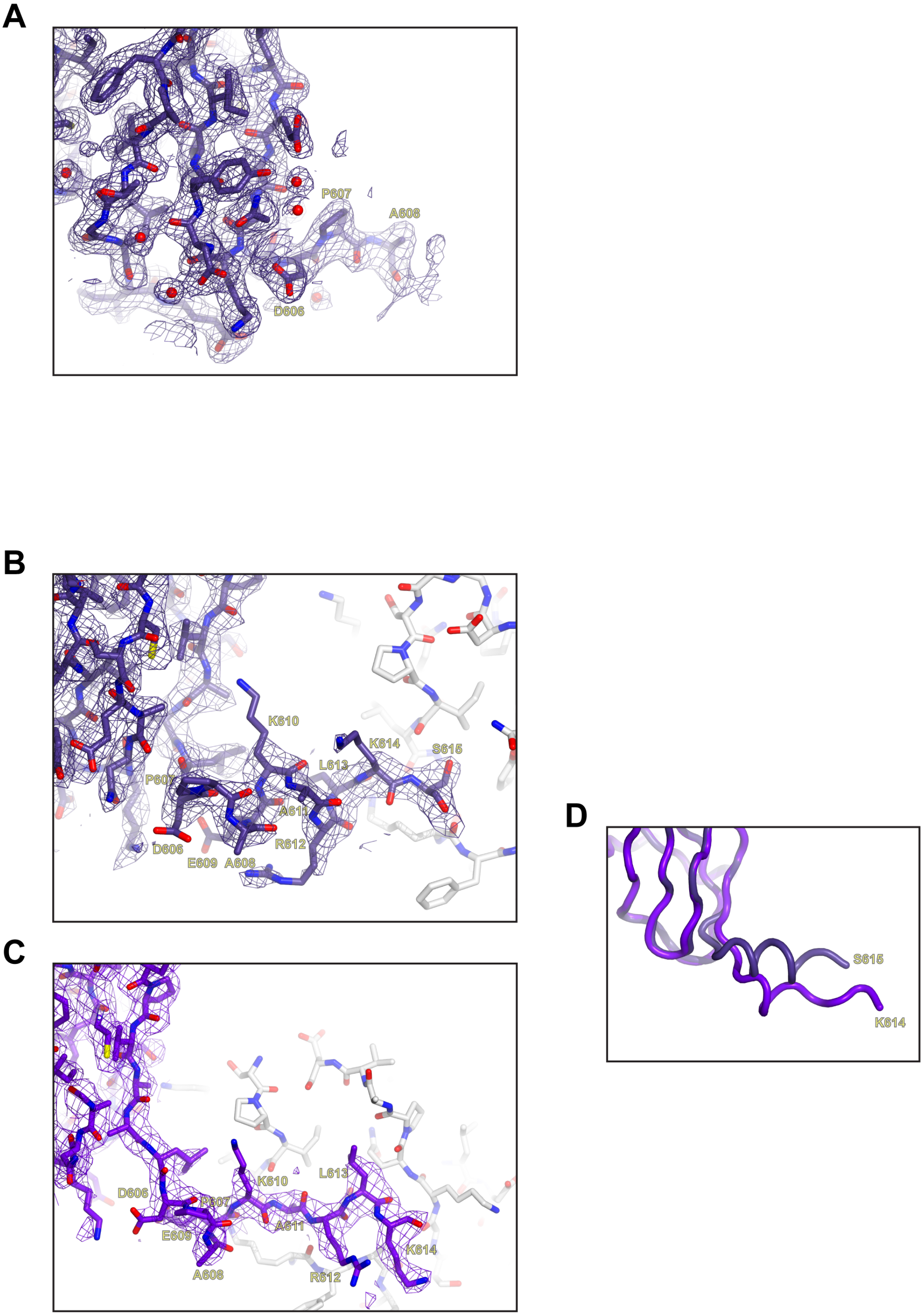
A
P607
A608
D606
B
K610
K614
S615
L613
P607
A611
D606
R612
E609
A608
C
L613
K610
D606
P607
A611
E609
K614
A608
R612
D
S615
K614

Figure S7 (related to Figure 5). The C-terminal helix is generally disordered in My4L_H (myomesin residues 510-618) crystals. (A-C) Close-up on the C-terminal region of My4L_H in space group $P6_5$ (2.05 Å resolution) (A) and in space group $P2_1$ (2.80 Å resolution) (B-C). $2mF_o$-DF_c electron density maps are shown in blue at the 1.0σ contour level. My4L_H models are shown as sticks in slate blue. Symmetry related molecules are in grey. Oxygen, nitrogen, sulphur atoms are in red, blue, yellow, respectively. In space group $P6_5$ all four My4L_H molecules in the a.u. display clear electron density until residue A608 while residues D609-S618 cannot be modelled (A). In space group $P2_1$ (six molecules/a.u) the L_H region folds into a helix in one molecule (B) whilst in another molecule this region is more extended (C). All other molecules display a disordered C-terminus that could not be modelled; (D) Cartoon tube superposition of My4L_H C-termini in helical and extended conformations.

Figure S8.

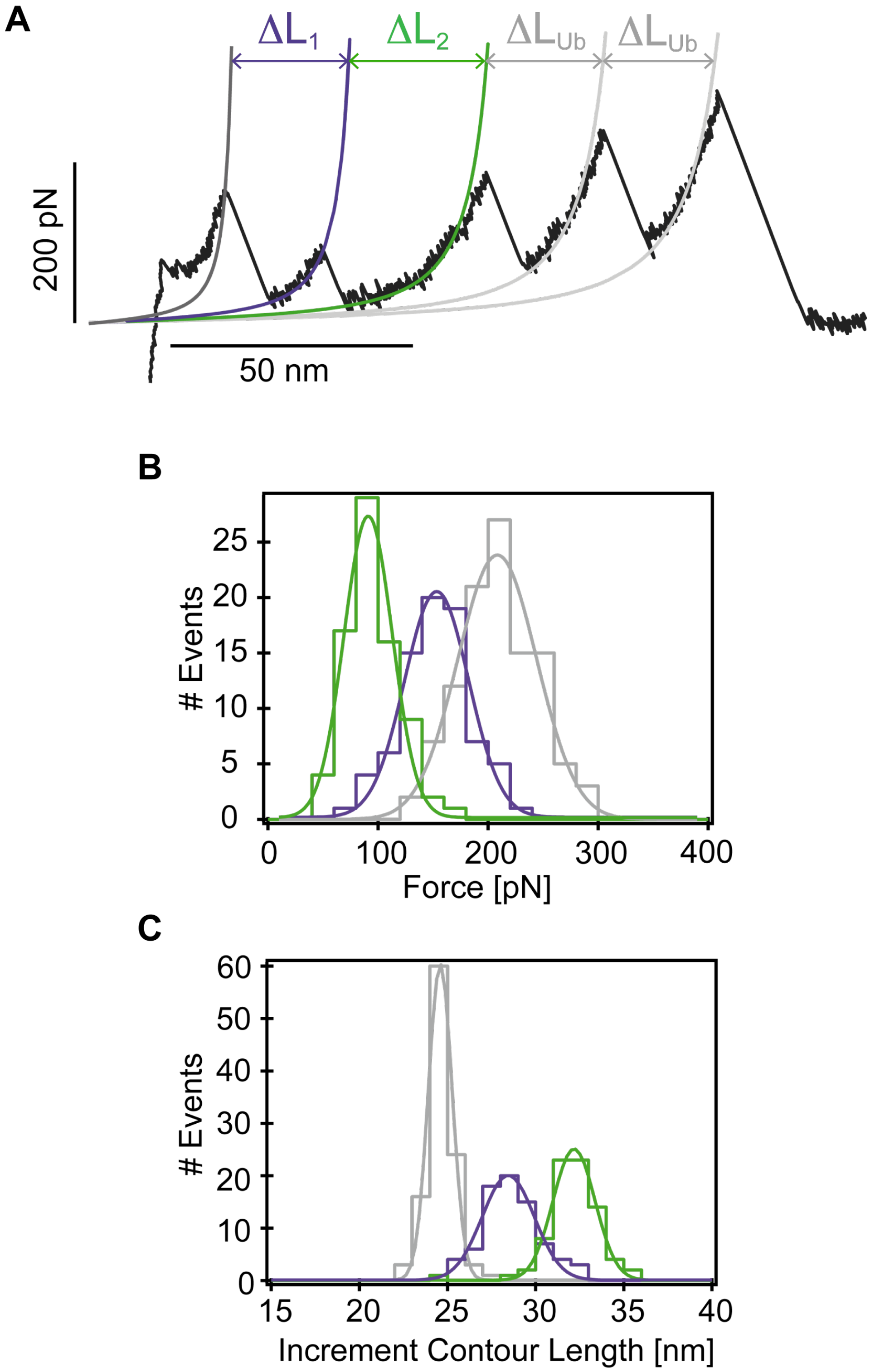

A
ΔL1
ΔL2
ΔLUb
ΔLUb
200 pN
50 nm
B
Events
25
20
15
10
5
0
0
100
200
300
400
Force [pN]
C
Events
60
50
40
30
20
10
0
15
20
25
30
35
40
Increment Contour Length [nm]

Figure S8 (related to Figure 6). Force spectroscopy measurements on the ubiquitin-*L*-(connector)$_{68}$-OL3-ubiquitin polyprotein. (A) Typical force-extension trace for the ubiquitin-*L*-(connector)$_{68}$-OL3-ubiquitin polyprotein. Fit to the worm-like chain model (WLC) provides contour length increases, ΔL, from single domains unfolding events; (B) Histogram of domain unfolding forces. Force peaks are estimated at 143±29 pN (n=78), 81±22 pN (n=78), and 198±35 (n=107), for *L* unlatching, OL3 and ubiquitin unfolding, respectively; (C) Histogram of measured contour length increases. ΔL_1=27.9±1.5 nm (n=78), ΔL_2=27.9±1.5 nm (n=78), and ΔL_{Ub}=23.8±0.6 nm (n=107), for *L* unlatching, OL3 and ubiquitin unfolding, respectively. In (B) and (C) colored curves are Gaussian fits.

Legend Movie S1 (related to Figure 5). Conformational transition from the compact conformation (as observed by SAXS) to the more extended geometry (as observed in the crystallographic dimer) for the OL3-My4*L* complex. My4*L* and OL3 are shown as surface representation in slate blue and green, respectively.

References

Bricogne, G., Blanc, E., Brandl, M., Flensburg, C., Keller, P., Paciorek, W., Roversi, P., Sharff, A., Smart, O.S., Vonrhein, C., *et al.* (2011). BUSTER version 2.10.0. Global Phasing Ltd., Cambridge, United Kingdom.

Emsley, P., Lohkamp, B., Scott, W.G., and Cowtan, K. (2010). Features and development of Coot. Acta Crystallogr. D, Biol. Crystallogr. *66*, 486-501.

Franke, D., and Svergun, D.I. (2009). DAMMIF, a program for rapid ab-initio shape determination in small-angle scattering. J. Appl. Crystallogr. *42*, 342-346.

Harpaz, Y., and Chothia, C. (1994). Many of the immunoglobulin superfamily domains in cell adhesion molecules and surface receptors belong to a new structural set which is close to that containing variable domains. J. Mol. Biol. *238*, 528-539.

Matthews, B.W. (1968). Solvent content of protein crystals. J. Mol. Biol. *33*, 491-497.

Mrosek, M., Labeit, D., Witt, S., Heerklotz, H., von Castelmur, E., Labeit, S., and Mayans, O. (2007). Molecular determinants for the recruitment of the ubiquitin-ligase MuRF-1 onto M-line titin. FASEB J. *21*, 1383-1392.

Murshudov, G.N., Skubak, P., Lebedev, A.A., Pannu, N.S., Steiner, R.A., Nicholls, R.A., Winn, M.D., Long, F., and Vagin, A.A. (2011). REFMAC5 for the refinement of macromolecular crystal structures. Acta Crystallogr. D, Biol. Crystallogr. *67*, 355-367.

Pernigo, S., Fukuzawa, A., Bertz, M., Holt, M., Rief, M., Steiner, R.A., and Gautel, M. (2010). Structural insight into M-band assembly and mechanics from the titin-obscurin-like-1 complex. Proc. Natl. Acad. Sci. USA *107*, 2908-2913.

Pernigo, S., Fukuzawa, A., Pandini, A., Holt, M., Kleinjung, J., Gautel, M., and Steiner, R.A. (2015). The crystal structure of the human titin:obscurin complex reveals a conserved yet specific muscle M-band zipper module. J. Mol. Biol. *427*, 718-736.

Petoukhov, M.V., Franke, D., Shkumatov, A.V., Tria, G., Kikhney, A.G., Gajda, M., Gorba, C., Mertens, H.D., Konarev, P.V., and Svergun, D.I. (2012). New developments in the program package for small-angle scattering data analysis. J Appl. Crystallogr. *45*, 342-350.

Popa, I., Kosuri, P., Alegre-Cebollada, J., Garcia-Manyes, S., and Fernandez, J.M. (2013). Force dependency of biochemical reactions measured by single-molecule force-clamp spectroscopy. Nat. Protoc. *8*, 1261-1276.

Schlierf, M., Li, H., and Fernandez, J.M. (2004). The unfolding kinetics of ubiquitin captured with single-molecule force-clamp techniques. Proc. Natl. Acad. Sci. USA *101*, 7299-7304.

Seeliger, D., Haas, J., and de Groot, B.L. (2007). Geometry-based sampling of conformational transitions in proteins. Structure *15*, 1482-1492.

Svergun, D.I. (1999). Restoring low resolution structure of biological macromolecules from solution scattering using simulated annealing. Biophys. J. *76*, 2879-2886.

Vagin, A., and Teplyakov, A. (2010). Molecular replacement with MOLREP. Acta Crystallogr. D, Biol. Crystallogr. *66*, 22-25.

Volkov, V.V., and Svergun, D.I. (2003). Uniqueness of ab initio shape determination in small-angle scattering. J. Appl. Crystallogr. *36*, 860-864.
