# Supplementary figures and images for: Binding of Myomesin to Obscurin-Like-1 at the Muscle M-Band Provides a Strategy for Isoform-Specific Mechanical Protection
Source: Structure. 2017 Jan 3;25(1):107–20. doi: 10.1016/j.str.2016.11.015 (PMC5222588; doi:10.1016/j.str.2016.11.015)

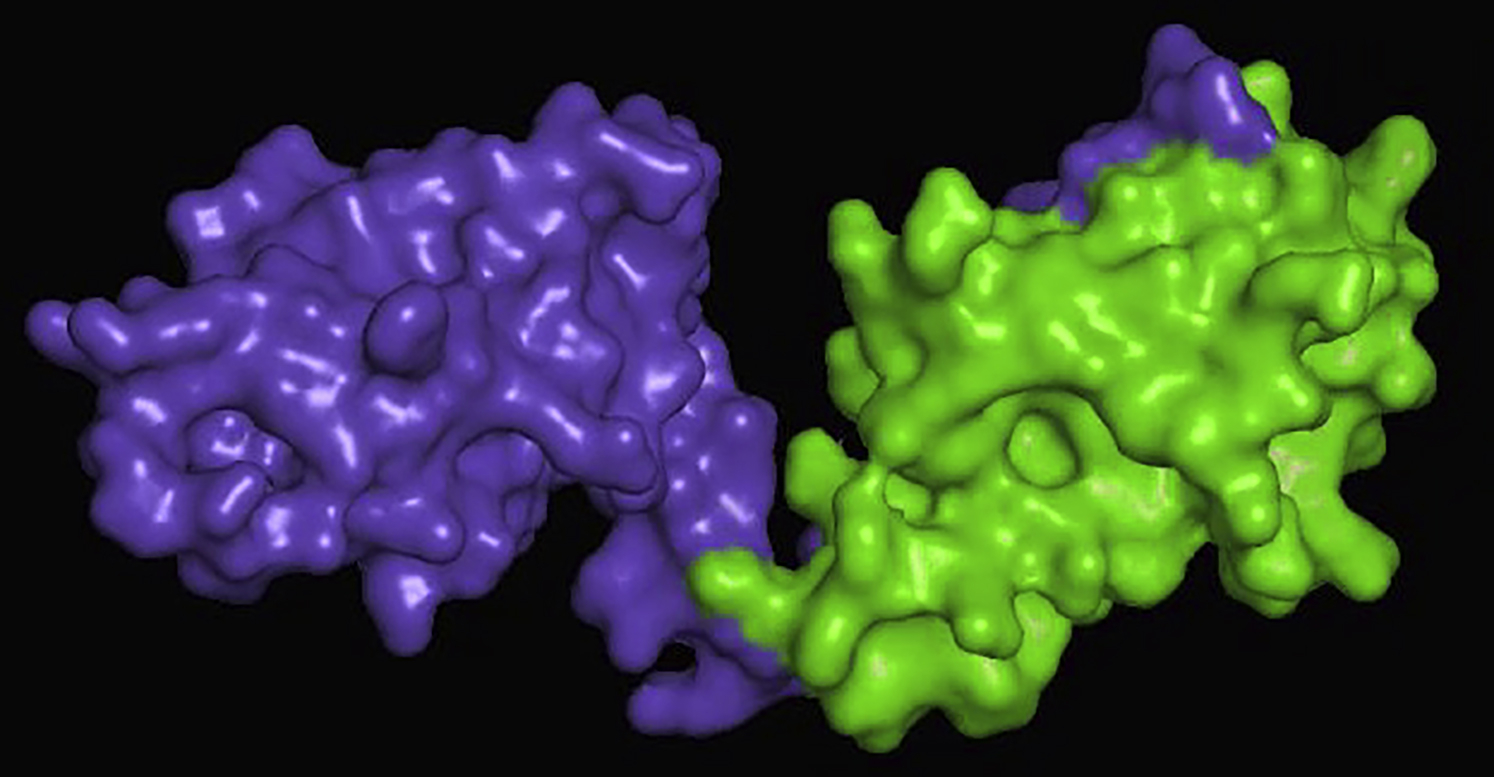

Supplement: Movie S1. Conformational Transition from the Compact Conformation as Observed by SAXS to the More Extended Geometry as Observed in the Crystallographic dimer for the OL3-My4L Complex, Related to Figure 5 [file mmc2.jpg]
