# Supplementary material for: Binding of Myomesin to Obscurin-Like-1 at the Muscle M-Band Provides a Strategy for Isoform-Specific Mechanical Protection
Source: Structure. 2017 Jan 3;25(1):107–20. doi: 10.1016/j.str.2016.11.015 (PMC5222588; doi:10.1016/j.str.2016.11.015)
Supplement: Document S2. Article plus Supplemental Information [file mmc3.pdf]

Structure

Article

Binding of Myomesin to Obscurin-Like-1 at the Muscle M-Band Provides a Strategy for Isoform-Specific Mechanical Protection

Graphical Abstract

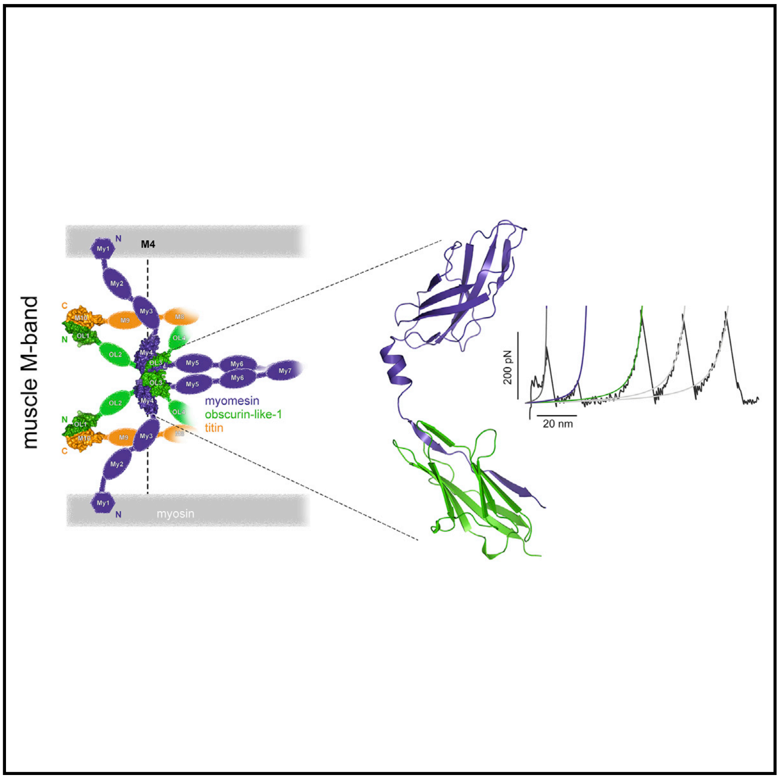

Highlights

- The structure of the human obscurin-like-1:myomesin complex has been determined
- A myomesin sequence complements an immunoglobulin fold of obscurin-like-1
- This binding mechanism provides mechanical stability up to forces of ∼135 pN
- Possible implications on muscle nanomechanics and M-band organization are discussed

Authors

Stefano Pernigo, Atsushi Fukuzawa, Amy E.M. Beedle, ..., Sergi Garcia-Manyes, Mathias Gautel, Roberto A. Steiner

Correspondence

sergi.garcia-manyes@kcl.ac.uk (S.G.-M.),
mathias.gautel@kcl.ac.uk (M.G.),
roberto.steiner@kcl.ac.uk (R.A.S.)

In Brief

Pernigo et al. analyze the myomesin-dependent integration of obscurin/obscurin-like-1 at the muscle M-band. They discover a mechanism of structural *trans*-complementation whereby an incomplete immunoglobulin-like domain of obscurin-like-1 assimilates an isoform-specific myomesin interdomain sequence providing mechanical stability.

Accession Numbers

5FM4
5FM5
5FM8

Pernigo et al., 2017, Structure *25*, 107–120
January 3, 2017 © 2016 The Author(s). Published by Elsevier Ltd.
http://dx.doi.org/10.1016/j.str.2016.11.015

CrossMark
CellPress

Structure
Article

Binding of Myomesin to Obscurin-Like-1 at the Muscle M-Band Provides a Strategy for Isoform-Specific Mechanical Protection

Stefano Pernigo,[1] Atsushi Fukuzawa,[1,2] Amy E.M. Beedle,[1,3] Mark Holt,[1,2] Adam Round,[4,6,7] Alessandro Pandini,[1,5] Sergi Garcia-Manyes,[1,3,*] Mathias Gautel,[1,2,*] and Roberto A. Steiner[1,8,*]
[1]Randall Division of Cell and Molecular Biophysics, King's College London, London SE1 1UL, UK
[2]Cardiovascular Division, King's College London BHF Centre of Research Excellence, London SE1 1UL, UK
[3]Department of Physics, King's College London, London WC2R 2LS, UK
[4]European Molecular Biology Laboratory, Grenoble Outstation, 38042 Grenoble, France
[5]Department of Computer Science and Synthetic Biology Theme, Brunel University London, London UB8 3PH, UK
[6]School of Chemical and Physical Sciences, Keele University, Keele, Staffordshire, UK
[7]Present address: Single Particles, Clusters, and Biomolecules and Serial Femtosecond Crystallography (SPB/SFX), European XFEL GmbH, Holzkoppel 4, 22869 Schenefeld, Germany
[8]Lead Contact
*Correspondence: sergi.garcia-manyes@kcl.ac.uk (S.G.-M.), mathias.gautel@kcl.ac.uk (M.G.), roberto.steiner@kcl.ac.uk (R.A.S.)
http://dx.doi.org/10.1016/j.str.2016.11.015

SUMMARY

The sarcomeric cytoskeleton is a network of modular proteins that integrate mechanical and signaling roles. Obscurin, or its homolog obscurin-like-1, bridges the giant ruler titin and the myosin cross-linker myomesin at the M-band. Yet, the molecular mechanisms underlying the physical obscurin(-like-1):myomesin connection, important for mechanical integrity of the M-band, remained elusive. Here, using a combination of structural, cellular, and single-molecule force spectroscopy techniques, we decode the architectural and functional determinants defining the obscurin(-like-1): myomesin complex. The crystal structure reveals a *trans*-complementation mechanism whereby an incomplete immunoglobulin-like domain assimilates an isoform-specific myomesin interdomain sequence. Crucially, this unconventional architecture provides mechanical stability up to forces of ~135 pN. A cellular competition assay in neonatal rat cardiomyocytes validates the complex and provides the rationale for the isoform specificity of the interaction. Altogether, our results reveal a novel binding strategy in sarcomere assembly, which might have implications on muscle nanomechanics and overall M-band organization.

INTRODUCTION

Sarcomeres, the basic contractile units of striated muscles, specialize in force generation through cyclic interactions of myosin and actin filaments. This fundamental activity requires the correct positioning of hundreds of proteins assembled in an overall functional architecture that need to respond to mechanical force in a cooperative, orchestrated way, as well as providing key integration of regulatory signals. The Z-disc and M-band sarcomeric regions (Figure 1A), although not directly involved in the actomyosin complex, are hubs where multiple structural and regulatory proteins are linked (Gautel and Djinovic-Carugo, 2016). In particular, the central M-band, where titin filaments entering from opposite half-sarcomeres overlap, has been proposed as a structural safeguard of sarcomere integrity during force-generation cycles (Agarkova et al., 2003).

Myomesin is a 185 kDa modular protein that localizes exclusively at the M-band, where anti-parallel dimers cross link myosin filaments (Figure 1B). It is expressed in all muscle types and its knockdown by siRNA results in a general failure in M-band assembly and the formation of disordered sarcomeres (Fukuzawa et al., 2008). Long interdomain α-helices at the protein's C-terminus have been suggested to act as strain absorbers enabling myomesin to buffer mechanical forces between molecules during muscle work (Pinotsis et al., 2012; Xiao and Grater, 2014). In addition to a mechanical role, myomesin is also needed for the integration of obscurin and its smaller obscurin-like-1 homolog at the M-band (Fukuzawa et al., 2008). Together with titin's C-terminus, a hotspot for disease-related mutations (Carmignac et al., 2007; Pollazzon et al., 2009), myomesin recruits obscurin and obscurin-like-1 N-termini at the myofibril periphery and core, respectively, establishing a ternary complex (Figure 1B).

Obscurin and obscurin-like-1 share a common immunoglobulin (Ig)-rich modular structure, which, in the case of obscurin, is more extended, featuring additional signaling and protein-binding domains absent in obscurin-like-1 (Fukuzawa et al., 2008). The presence of a non-modular C-terminus able to interact with small ankyrin-1 isoform 5 and ankyrin-2 led to the suggestion that obscurin plays a role in establishing the sarcomere-sarcoplasmic reticulum connection (Bagnato et al., 2003; Kontrogianni-Konstantopoulos et al., 2003). The pathophysiological roles of these proteins are only beginning to emerge. Ablation of obscurin in mice results in changes in longitudinal sarcoplasmic reticulum architecture with alterations in several SR-associated proteins (Lange et al., 2012) as well as marked

This is an open access article under the CC BY license (http://creativecommons.org/licenses/by/4.0/).

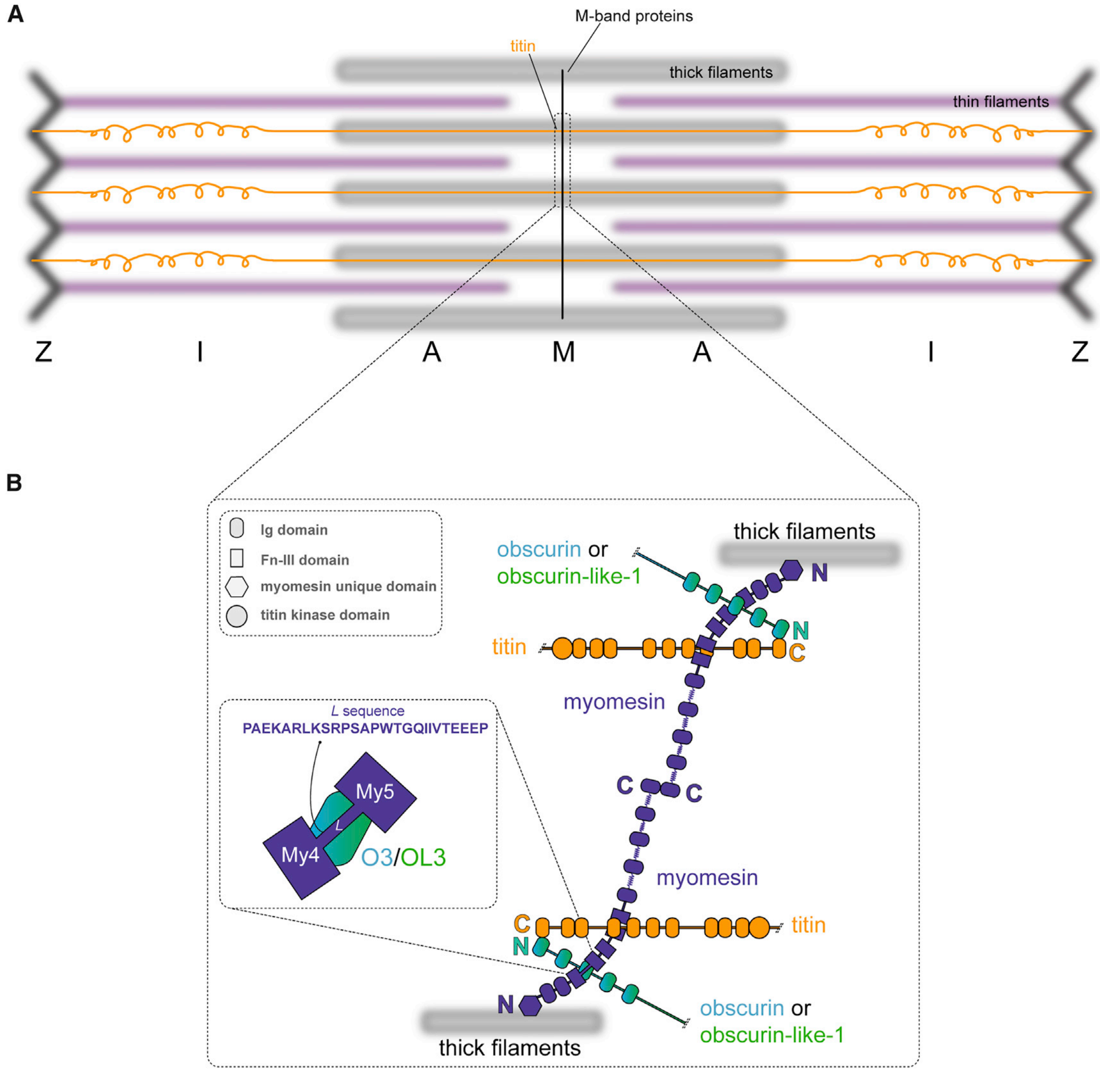

Figure 1. Schematic of the M-Band Network

(A) Principal sarcomere regions are marked by the letters Z, I, A, and M.

(B) Modular myomesin, titin, and obscurin/obscurin-like-1 proteins form an intricate M-band network with C-terminal myomesin dimers crosslinking myosin filaments. The inset highlights the interaction between myomesin and obscurin/obscurin-like-1, which has been mapped to linker sequence (*L*) located between the myomesin fibronectin (Fn-III) domains My4 and My5 and the third immunoglobulin (Ig) domain of obscurin/obscurin-like-1 (O3/OL3, respectively) (Fukuzawa et al., 2008).

sarcolemma fragility and reduced muscle exercise tolerance (Randazzo et al., 2013), while its depletion in zebrafish leads to disturbances in the extracellular matrix organization during skeletal muscle development (Raeker and Russell, 2011). The founding member of the obscurin family of proteins is UNC-89 in *Caenorhabditis elegans* (Benian et al., 1996). *unc-89* loss-of-function mutant worms display reduced locomotion, disorganized myofibrils, and lack M lines (Small et al., 2004; Waterston et al., 1980). *unc-89* mutants show disorganization of myosin thick filaments by immunostaining (Qadota et al., 2008; Wilson et al., 2012). *Drosophila* expresses a protein more similar to nematode UNC-89 than to vertebrate obscurin. In *Drosophila,* RNAi experiments indicate that obscurin is needed for the formation of normal symmetrical sarcomeres (Katzemich et al., 2015). However, fundamental differences exist in the domain patterns and likely functions of the signaling domains in vertebrate, insect, and nematode obscurins/unc-89 members. All obscurin/UNC-89 members contain a constitutively expressed Rho-type GDP/GTP exchange factor domain (GEF) with a preceding Src-homology-3 (SH3) domain, which in insect and nematode obscurin/UNC-89 are situated at the N-terminal end of the proteins, while in vertebrate obscurin, the GEF domain is at the C-terminus. In addition, obscurin/UNC-89 isoforms can contain up to two serine/threonine kinase domains (Katzemich et al., 2012; Spooner et al., 2012). In insect and nematode obscurin, these are catalytically inactive pseudokinases that form scaffolds for the interactions with regulators of sarcomere assembly and/or maintenance (Katzemich et al., 2012), while the two differentially spliced kinases in vertebrate obscurin contain all canonical residues required for catalysis (Fukuzawa et al., 2005) and

Table 1. X-Ray Data Collection and Refinement Statistics

Data Collection			
Dataset	OL3:My4*L*	My4L_H (1)	My4L_H (2)
Beamline	I04 (DLS)	I04 (DLS)	I04-1 (DLS)
Wavelength (Å)	0.9778	0.97949	0.92819
Resolution range[a] (Å)	67.19–3.10 (3.18–3.10)	84.36–2.05 (2.11–2.05)	39.21–2.80 (2.87–2.80)
Space group	*C*2	*P*6_5	*P*2_1
Cell dimensions			
(a, b, c) (Å)	91.18, 134.38, 68.39	97.41, 97.41, 106.15	43.53, 156.84, 48.26
(α, β, γ) (°)	90, 92.50, 90	90, 90, 120	90, 94.38, 90
Unique reflections[a]	14,811 (1,064)	35,371 (2,587)	15,138 (1,048)
Overall redundancy[a]	4.5 (4.5)	20.0 (20.0)	3.1 (3.1)
Completeness[a] (%)	99.1 (98.2)	99.9 (98.7)	95.3 (89.8)
R_{merge}[a] (%)	9.8 (69.8)	13.4 (242.1)	9.0 (31.4)
$R_{p.i.m.}$ (I)[a] (%)	6.2 (44.3)	3.6 (56.7)	8.1 (24.5)
$\langle I/\sigma(I)\rangle$[a]	12.7 (1.8)	13.4 (1.6)	10.8 (3.1)
**Refinement**			
PDB code	5FM5	5FM8	5FM4
R_{factor} (%)/R_{free} (%)	21.6/25.9	19.2/21.7	26.1/28.7
Twin/twin fraction	no	k,h,−l/0.67	no
No. of non-H atoms			
Protein	3,482	3,142	4,726
Water	84	140	37
Average *B* value (Å^2)	85.5	50.1	35.2
Root-mean-square bond lengths (Å)	0.009	0.013	0.005
Root-mean-square bond angles (°)	1.46	1.72	1.07

[a]Numbers in parentheses refer to the highest resolution bin.

were reported to be catalytically active in vitro (Hu and Kontrogianni-Konstantopoulos, 2013). Analyzing the molecular interactions and signaling functions therefore requires dedicated approaches for each of these presumptive homologs. From a pathological viewpoint, obscurin polymorphisms has been linked to hypertrophic cardiomyopathy (Arimura et al., 2007) and dilated cardiomyopathy (Marston et al., 2015), while mutations in obscurin-like-1 have been linked to the rare hereditary growth retardation 3-M syndrome (Huber et al., 2009) with a role in the maintenance of cullin-7 levels (Hanson et al., 2009). Understanding obscurin/UNC-89 functions thus also bears relevance to understanding the impact of pathogenic variants in human.

To advance knowledge on M-band organization and function, we have previously established the molecular basis for titin:obscurin-like-1 (Pernigo et al., 2010) and titin:obscurin (Pernigo et al., 2015) connection at the M-band. Obscurin and obscurin-like-1 use their homologous N-terminal immunoglobulin-like (Ig) domains (O1 and OL1, respectively) to bind titin's most C-terminal Ig domain (M10) in a mutually exclusive manner and in a unique chevron-shaped anti-parallel Ig-Ig architecture (Pernigo et al., 2010, 2015; Sauer et al., 2010). Mechanically, the M-band titin:obscurin(-like-1) junction is labile, as in single-molecule force spectroscopy experiments both M10:O1 and M10:OL1 complexes yield at forces of around 30 pN (Pernigo et al., 2010). An obvious missing piece in the M-band structural puzzle is the molecular architecture of the obscurin(-like-1):myomesin complex, a key elusive element to understand the global geometry and mechanical stability defining the M-band. Using a multidisciplinary approach encompassing structural techniques, in vivo cellular competition assays, and single-molecule force spectroscopy experiments, we investigated here the myomesin-dependent mechanism of obscurin(-like-1) integration at the M-band.

RESULTS

Human Obscurin/Obscurin-like-1:Myomesin Complex for Structural Analysis

Large muscle proteins are typically modular, featuring several Ig and fibronectin-type-III (Fn-III) domains interspersed by linkers of variable length and structural order. Yeast two-hybrid and biochemical analyses have mapped the obscurin/obscurin-like-1:myomesin interaction to the linker region (*L*) located between the fourth and fifth Fn-III domains of myomesin (My4 and My5, respectively) and the third Ig domain of either obscurin or obscurin-like-1 (O3 and OL3, respectively) (Figure 1B, inset) (Fukuzawa et al., 2008). O3 and OL3 are highly homologous, sharing 47.2% sequence identity. To produce protein complexes for structural analysis, we initially attempted the expression of isolated domains in *Escherichia coli,* but failed to obtain soluble O3 or OL3. We therefore decided to pursue a co-expression approach and cloned either O3 or OL3 C-terminal to a GST tag in the first expression cassette of a bicistronic vector, where the myomesin region encompassing the fourth and fifth Fn-III domains (My4*L*My5) was cloned in the second expression cassette. This strategy readily produced soluble protein for both constructs and size-exclusion chromatography (SEC) analysis of GST-cleaved complexes is consistent with the formation of obscurin(-like-1):myomesin heterodimers with a 1:1 stoichiometry (Figure S1). Using a matrix microseeding (MMS) approach (D'Arcy et al., 2014), we successfully crystallized the OL3:My4*L*My5 complex and solved its structure at the 3.1 Å resolution. X-ray data collection and refinement statistics are given in Table 1. The final model is characterized by excellent statistics and R/R_{free} (%) values of 21.6/25.9. While the myomesin My4 domain and its C-terminal linker *L* as well as obscurin-like-1 OL3 are well defined in the structure, the entire myomesin My5 domain is invisible in electron density maps. Proteolysis during crystallization does not appear to be the reason for this, as SDS-PAGE analysis of dissolved crystals shows both My4*L*My5 and OL3 components at the expected molecular weight (Figure S2). Thus, we conclude that the lack of electron density for My5 is due to its positional disorder in the crystal. As My5 is not visible in the structure, we hereafter refer to the crystallographic complex as to OL3:My4*L*.

Overall Organization

The OL3:My4*L* complex is present as a (OL3:My4*L*)$_2$ dimer of heterodimers in the crystal (Figure 2A). Individual OL3:My4*L* complexes display a bent dumbbell-shaped structure, in which

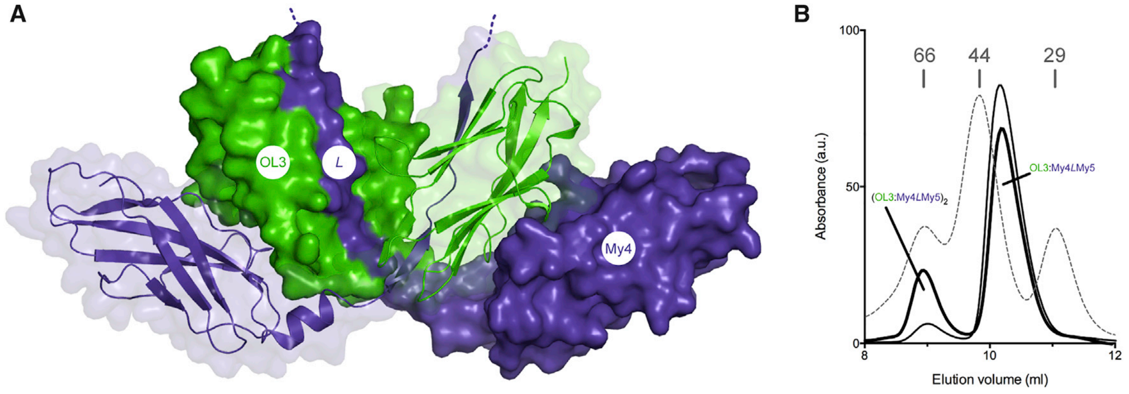

Figure 2. The (OL3:My4*L*)$_2$ Complex Dimer

(A) In the crystal, two OL3:My4*L* heterodimers are arranged around a non-crystallographic two-fold axis (vertical in this view) forming a W-shaped (OL3:My4*L*)$_2$ dimeric assembly. My4*L* and OL3 are in slate blue and green, respectively, with one heterodimer shown as a solid surface and the other in cartoon mode with a transparent surface. The dotted line at the C-terminus of *L* indicates the flexible region leading to My5 not visible in the structure.

(B) Superdex S75 10/300 SEC traces for the OL3:My4*L*My5 complex injected in the column at 3 mg/mL (thin continuous line) and 8.2 mg/mL (thick continuous line). The OL3:My4*L*My5 complex forms a mixture of monomers and dimers in a concentration-dependent manner. The elution profile of calibration markers is shown with a gray broken line. Molecular weights are indicated.

the myomesin *L* linker extends away from the My4 domain integrating within the OL3 fold. Two OL3:My4*L* heterodimers then interlock around a non-crystallographic two-fold axis, giving rise to a dimeric assembly with overall dimensions of 105 Å × 48 Å × 26 Å. Large solvent channels running parallel to the molecular dyad axis are observed in the crystallographic packing (Figure S3). These are compatible with the presence of positionally disordered My5 domains.

As the OL3:My4*L*My5 complex typically elutes from SEC as a monomeric unit during purification (Figure S1), we analyzed its behavior at concentrations similar to that used for crystallization. In the accessible range of 3.0–8.2 mg/mL (0.082–0.225 mM), we observed the formation of complex dimers in a concentration-dependent manner with an approximately 30:70 dimer:monomer ratio at the highest protein concentration (Figure 2B). Thus, the oligomerization state observed in the crystal reflects that of a population in solution promoted by high protein concentration (~15.9 mM in the crystal).

The OL3:My4*L* Heterodimer

Three distinct structural regions that we identify as My4, L_H, and OL3$_A$ contribute to the architecture of individual OL3:My4*L* heterodimers (Figures 3A and 3B). The My4 domain displays the typical Fn-III fold made of seven anti-parallel β-strands organized in two separate β-sheets (A-B-E and C-C′-F-G) arranged in a β sandwich. C′ is rather short, while G is broken in two (G′ and G″) and interacts with the beginning and end of the long F β-strand. C-terminal to My4, the L_H spacer region encompasses the first 11 amino acids of *L* and is formed by a 10.6-Å-long α-helix (Pro^{607}-Lys^{614}) followed by a short three amino acids peptide (Ser^{615}-Pro^{617}). L_H leads the C-terminal portion of *L,* an 18-amino-acid-long extended stretch divided into two β-strands ($L_{S'}$ and $L_{S''}$) that integrate within the OL3 Ig fold. Similar to the Fn-III architecture, the Ig fold is also organized into a β sandwich formed by two β-sheets (A′-G-F-C-C′ and $L_{S'}$/$L_{S''}$-B′/B″-E-D). As OL3 integrates structural elements of *L,* we refer to this portion of the complex as the augmented OL3 (OL3$_A$) domain. The distinctively bent geometry of the heterodimer is dictated by the principal axes of My4 and OL3$_A$ forming a ~100° angle along the longest dimension of the complex. This, coupled with the 18.2-Å-long L_H region (Pro^{607}-Pro^{617}, Cα-Cα distance) that acts as a spacer between the domains, allows for the positioning of the second OL3:My4*L* complex within the tetrameric assembly (Figure 2A).

OL3$_A$ is an example of fold complementation (Figure 3C), and the isolated OL3 domain is best described as an incomplete Ig of the intermediate-set (I-set) subfamily (Harpaz and Chothia, 1994). This type of Ig is often found in muscle proteins (Otey et al., 2009) and consists of a total of nine strands arranged into two distinct β-sheets (A-B-E-D and A′-G-F-C-C′), exhibiting the characteristic discontinuous A/A′ strand distributed over both β-sheets (Figure S4A). In OL3, the A β-strand that is hydrogen-bonded to B is missing and is replaced by myomesin $L_{S'}$ (Ser^{618}-Thr^{622}), thus re-establishing a complete Ig architecture. A second myomesin strand ($L_{S''}$, Ile^{626}-Glu^{630}) also hydrogen bonds to B″ at a position that is reminiscent of the A′ positioning found in a few deviant I-set Ig domains, identified as the I*-set subtype (Pernigo et al., 2015). Members of this subtype feature a relocation of their A′ strand, resulting in the formation of an A/A′-B-E-D β-sheet (Figure S4B). Thus, OL3$_A$ is a complex *trans*-complemented hybrid I/I*-set Ig fold.

Molecular Interfaces

Two sets of molecular interfaces are present in the crystallographic structure. The first one is involved in the formation of the OL3:My4*L* heterodimer. An additional set of interactions enables its dimerization. As SEC analysis indicates that in solution the formation of the (OL3:My4*L*)$_2$ assembly is promoted by high concentration of the complex (Figure 2B), this implies that homodimerization is hierarchically secondary to the establishment of the OL3:My4*L* interface.

As highlighted in the contact maps in Figures 3D and 3E, the OL3:My4*L* heterodimer is held together by Ig-fold complementation. A mixture of hydrogen bonds and hydrophobic interactions stabilizes the heterodimer (Figure 3F). One edge of the

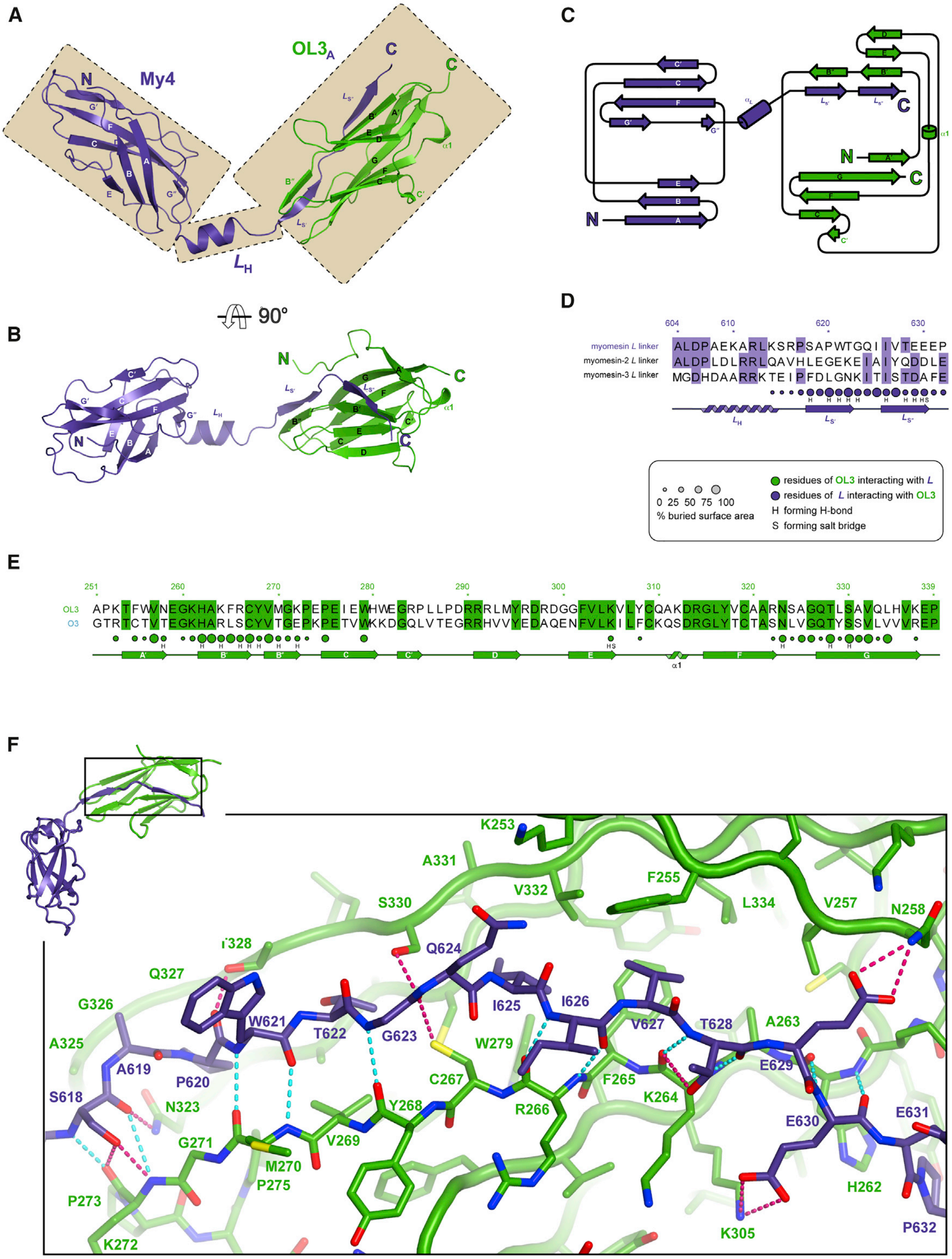

Figure 3. OL3:My4*L* Heterodimer

(A and B) Cartoon representation of the OL3:My4*L* heterodimer. The view in (B) is rotated by 90° around the *x* axis compared with (A). My4*L* and OL3 are shown in slate blue and green, respectively. In (A), the three main regions contributing to the complex are highlighted: the My4 Fn-III domain, its C-terminal helical spacer L_H, and the $OL3_A$ (A for augmented) domain in which the Ig OL3 domain is stabilized by myomesin fold complementation in-*trans*.

(C) Topology diagram of the complex.

(legend continued on next page)

mixed $L_{S'}/L_{S''}$-B′/B″-E-D β-sheet is engendered by the anti-parallel pairing of $L_{S'}$-B″ and $L_{S''}$-B′ β-strands mediated by a total of 11 main-chain hydrogen bonds (in cyan in Figure 3F) connecting $L_{S'}/L_{S''}$ residues to B′/B″ residues. Side chains also stabilize the complex by hydrogen bonding (in pink in Figure 3F). They typically involve hydroxyl groups of Thr and Ser residues (myomesin S618, T628 and OL3 T328, S330) interacting with main-chain carbonyl oxygen atoms. A single salt bridge connects the carboxylate of myomesin E630 to the amine side chain of OL3 K305. A number of hydrophobic residues are buried upon complex formation. For example, myomesin I625 points its aliphatic side chain in a tight cavity lined by OL3 F265, W279, L304, Y317, C319, V332. Together with myomesin P620, T622, and V627, this residue buries more than 90% of its surface in the interaction, representing a critical determinant for binding. Overall, the OL3:My4*L* interface area is 1,046 Å^2.

OL3:My4*L* homodimerization further stabilizes the assembly, resulting in the establishment of an interface area of 2,320 Å^2 (Figure S5). This is largely engendered by OL3*:My4*L* (and symmetric OL3:My4*L**, where * indicates that the domain belongs to the dimer partner), while My4*L*:My4*L** and OL3:OL3* interactions are rather limited with interface areas of 122 Å^2 and 278 Å^2, respectively. PISA analysis (Krissinel and Henrick, 2007) indicates positive ΔG dissociation values of 14.5 kcal/mol and 12.75 kcal/mol for (OL3:My4*L*)$_2$ and OL3:My4*L* stable assemblies, respectively.

Molecular Basis for Myomesin Isoform Specificity

The myomesin gene family comprises three *MYOM* genes in humans (Schoenauer et al., 2008). *MYOM1* encodes the ubiquitously expressed myomesin protein, while *MYOM2* and *MYOM3* encode a fast-fiber isoform called M-protein or myomesin-2 and myomesin-3, a recently identified isoform of slow fibers, respectively. The interaction with obscurin/obscurin-like-1 is limited to myomesin, as neither M-protein nor myomesin-3 shows any appreciable binding (Fukuzawa et al., 2008). Our X-ray structure explains the molecular basis for this specificity. Three myomesin residues mapping onto the *L* linker (T622, I625, and V627) display side chains that are complementary to the OL3 surface (Figure 4A). These are not conserved in either M-protein or myomesin-3 and occasionally exhibit rather dramatic amino acid substitutions. For example, myomesin T622 is replaced by a lysine in M-protein, while in myomesin-3 a more polar threonine takes the place of myomesin I625 (Figure 3E).

To validate the interaction between myomesin and obscurin/obscurin-like-1 in the context of the sarcomere, we generated a number of myomesin variants targeting the *L* linker and tested them for their ability to compete endogenous obscurin from the M-band. A quantitative analysis of these results is summarized in Figure 4B, while immunofluorescence images of representative experiments are shown in Figures 4C–4G and S6. When overexpressed in neonatal rat cardiomyocytes (NRCs), GFP-My4*L*My5 targets the M-band, in addition to other diffuse subcellular localizations, displacing endogenous obscurin (first bar in Figures 4B and 4C). In the case of T622, its replacement with an isosteric valine (T622V) does not significantly alter the wild-type behavior (second bar in Figures 5B and 4D). This is consistent with the lack of hydrogen bonding between the side chain of T622 and OL3 residues contributing to the small receptor cavity (Figures 3F and 4A). However, when T622 is replaced by a lysine (T622K) as in M-protein (third bar in Figures 4B and 4E), or alternatively when I625 is replaced by a threonine like in myomesin-3 (fourth bar in Figures 4B and 4F), competition is essentially abrogated. A similar effect is mediated by the V627Y replacement also found in M-protein (fifth bar in Figures 4B and 4G). As expected, control substitutions targeting myomesin regions not involved in the interface have no effect on the ability to compete endogenous obscurin (Figure S6).

The OL3:My4*L* Heterodimer Is a Flexible Structural Element

The bent dumbbell shape of OL3:My4*L* observed in the crystal is stabilized by its homodimeric assembly. As SEC analysis indicates that the complex is predominantly monomeric in solution, we explored whether this geometry is representative of the complex in solution using small-angle X-ray scattering (SAXS). The overall molecular parameters derived from scattering data on OL3:My4*L* and OL3:My4*L*My5 are shown in Figure 5A. A comparison of the experimental radius of gyration R_g for OL3:My4*L* (25.2 ± 2 Å) with that calculated from the structure (28.9 Å) indicates that in solution, the complex adopts a less extended conformation than in the crystal. Accordingly, the scattering pattern computed from the crystallographic model yielded a suboptimal fit (χ = 1.91) to the SAXS data (Figure 5B, upper curve, blue line), suggesting differences in the relative domain arrangement. To investigate the structure in solution, we considered the complex composed of three rigid bodies defined by the My4, L_H, and OL3$_A$ structural regions (Figure 3A). A good fit to the scattering curve was obtained with a model that is more compact than that seen in the crystal. We then used this structure as a starting template and, following energy minimization, generated >30,000 additional models (a selection shown in Figure 5C) using the *tCONCOORD* (Seeliger et al., 2007) algorithm, a computationally efficient method for sampling conformational transitions. Within this large pool, we found ~500 models that provide an excellent fit (χ < 1.0) to the experimental curves (Figure 5B, upper panel, red line). These models all display the L_H helix resting on the OL3$_A$ domain, resulting in a less extended conformation compared with the dimer-stabilized crystal structure (a selection shown in Figure 5D). Additional SAXS data measured on OL3:My4*L*My5 reveal that inclusion of the My5 domain increases the R_g value to 31.0 ± 2 Å (Figure 5A). To model this complex, we started from the OL3:My4*L* solution model and

(D and E) Sequence alignment of the linker region for the different myomesin isoforms (D) and of the OL3 and O3 domains (E). Identical amino acids are highlighted. Colored circles above the secondary structure cartoons show residues of one domain contacting the other as indicated in the inset. Their radius is proportional to the buried area. The letter codes H and S indicate residues involved hydrogen bonds and salt bridges, respectively.

(F) Enlarged stick representation of the boxed region in the top left-hand corner (view rotated approximately 90° around the *x* axis compared with B) highlighting important myomesin:obscurin-like-1 interactions. Nitrogen, oxygen, and sulfur atoms are shown in blue, red, and yellow, respectively. Hydrogen bonds involving main-chain atoms only are shown as dotted cyan lines. H bonds and salt bridges involving side chains are indicated by dotted magenta lines.

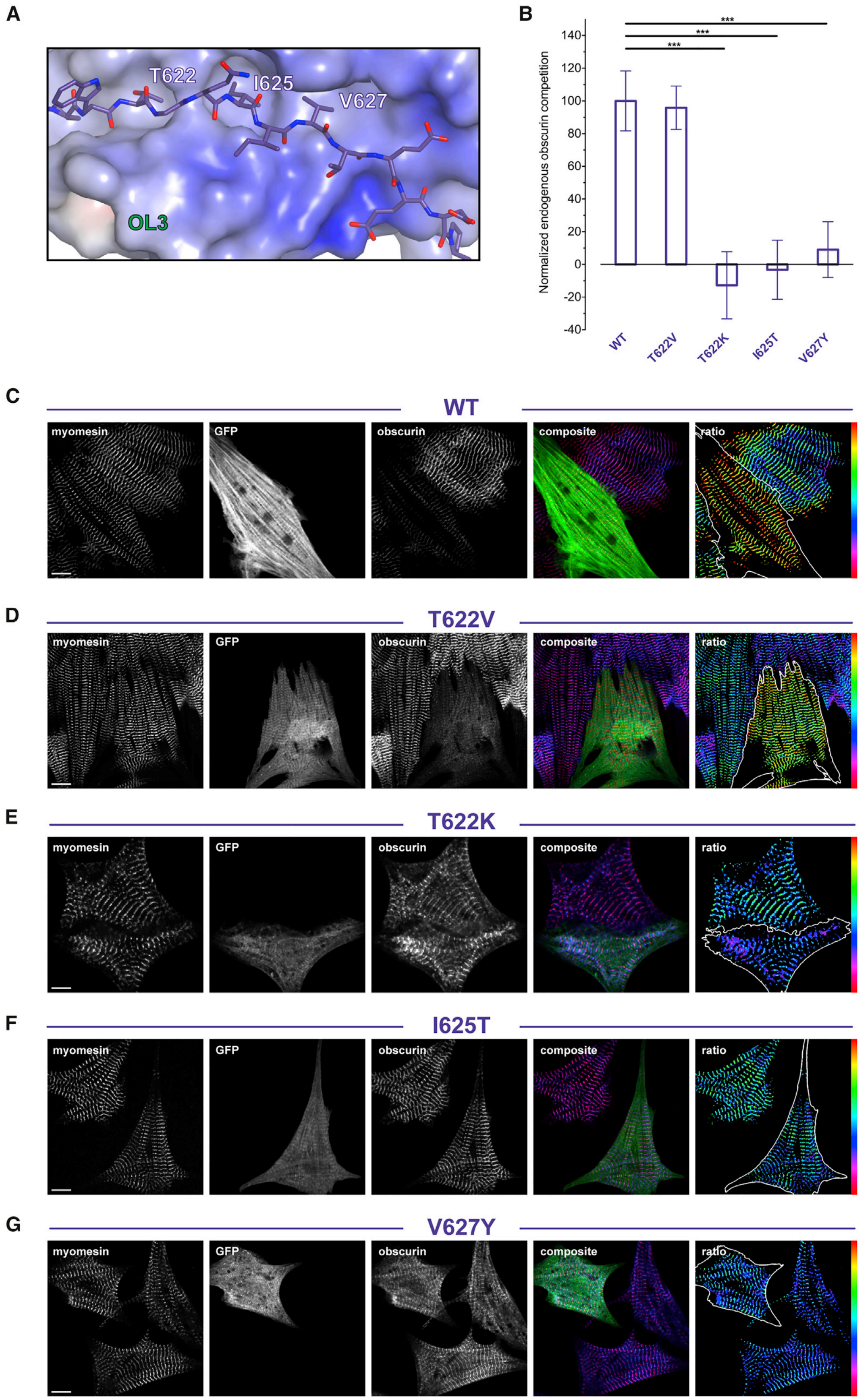

(legend on next page)

again used *tCONCOORD* to sample the conformational space following addition of an additional Ig domain (My5). Several similar models provide an excellent fit ($\chi < 1.0$) to the scattering curve (Figure 5B, lower curve). We find that the OL3:My4*L* portion of the complex remains largely invariant, with My5 approximately orthogonal to $OL3_A$ (Figure 5F).

The ability of OL3:My4*L* to transition from the solution conformation to that observed crystallographically suggests that the L_H helix might have a degree of flexibility. We explored this by solving the crystal structure of My4L_H (myomesin residues 510–618) in two different space groups (data collection and refinement statistics in Table 1). In space group $P6_5$ (2.05 Å resolution), all four My4L_H independent molecules in the a.u. display clear electron density until residue A608, while residues E609-S618 (L_H) cannot be modeled (Figure S7A). The same applies for four of six My4L_H independent molecules in the alternative $P2_1$ space group (2.80 Å resolution). However, in the latter crystal structure, crystal contacts stabilize the C-terminal region in the remaining two other My4L_H molecules. While in one molecule, L_H folds into an α-helix as in the My4*L*:OL3 complex (Figure S7B), in the other molecule the C-terminus is in a more extended conformation (Figure S7C). Overall, SAXS and crystallographic analyses support a model in which interdomain freedom allows the transition (see Movie S1) from a relatively compact solution conformation to an open one that can be stabilized by homodimerization.

Mechanical Stability of the Complex

It is enticing to speculate that the physical connection via swapped secondary structure elements might act as the molecular glue necessary for the mechanical stability of the obscurin(-like-1):myomesin assembly. To probe this, we employed single-molecule force spectroscopy using atomic force microscopy (AFM), and guided by the structure, we fused the C-terminus of the myomesin *L* linker to the N-terminus of OL3 by an unstructured 43 amino acids connector. This single-chain *L*-(connector)$_{43}$-OL3 complex was then sandwiched between two ubiquitin (Ub) domains that serve as well-characterized handles (Carrion-Vazquez et al., 2003) (Figure 6A). The engineered polyprotein enables the unambiguous characterization of the forces required to break the molecular interactions that hold the complex together.

When stretched in our AFM setup at the constant velocity of 400 nm s^{-1} often employed in these types of studies (del Rio et al., 2009; Garcia-Manyes et al., 2012; Perez-Jimenez et al., 2006), the polyprotein unfolded displaying a saw-tooth pattern with peaks of alternating mechanical stability (Figure 6B). At the beginning of the trace, we identified two mechanical events with associated contour length increments of ΔL_1 = 20.2 ± 1.2 nm (*n* = 66) and ΔL_2 = 31.0 ± 0.9 nm (*n* = 68), respectively, followed by the unfolding of the two ubiquitin monomers (ΔL_{Ub} ~24.5 nm), which serve as internal molecular calibration fingerprints (Figure 5B). Interestingly, the observed unfolding pattern does not follow the expected hierarchy of mechanical stability (Li and Fernandez, 2003). The first event occurs at a force value of 129.4 ± 27.0 pN (*n* = 66) while the second one only requires 86.6 ± 29.1 pN (*n* = 68) (Figure 5C). Both mechanical events are followed by the unfolding of the two Ub monomers, occurring at a higher force ~200 pN (Carrion-Vazquez et al., 2003). Such unfolding scenario is hence reminiscent of a molecular mechanism whereby a mechanically labile domain is mechanically protected from the pulling force by a more resilient protein structure (Peng and Li, 2009).

The crystal structure shows that a stretch of 15 amino acids belonging to *L* lies within the OL3 domain. As the engineered protein connector is 43 residues long, the predicted length increase as a result of *L* detachment from OL3 is ΔL_1=((15 + 43) residues × 0.36 nm/residue) = 20.88 nm. This value is in agreement with the experimental measurement (ΔL_1 = 20.2 ± 1.2 nm, Figure 5D). The second unfolding event (ΔL_2 = 31.0 ± 0.9 nm) corresponds to the unfolding and stretching of OL3 (89 amino acids). Thus, the single-molecule unfolding trajectories support an unfolding scenario whereby the first high-force event corresponds to the removal of the *L* linker from the OL3 domain, followed by the unfolding of OL3, occurring at a significantly lower force. To further confirm our molecular hypothesis, we constructed a second polyprotein in which the flexible connector length was lengthened to 64 residues. This new construct confirmed forces of 143 ± 29 pN (ΔL_1 = 27.9 ± 1.5 nm) and 81 ± 22 pN (ΔL_2 = 31.7 ± 1.2), for the detachment of the *L* latch and OL3 unfolding, respectively. As expected, while ΔL_2 is invariant in the two polyproteins, the longer ΔL_1 is fully consistent with the predicted extension of 28.4 nm ((15 + 64) residues × 0.36 nm/residue) for the longer connector (Figure S8). Our single-molecule nanomechanical experiments thus unambiguously support a molecular organization in which the mechanically labile OL3 domain is protected from force by a more resilient architecture afforded by myomesin *L* complementation (Peng and Li, 2009).

DISCUSSION

The reason why muscle sarcomeres do not self-destruct during contraction lies in the intricate yet poorly understood cytoskeletal protein networks coordinated by titin at the Z-disk and M-band, which link actin and myosin filaments transversally and longitudinally (Horowits et al., 1986). The M-band network

Figure 4. Cellular Validation and Myomesin Isoform Specificity

(A) Close up of the OL3:My4*L* interface. OL3 is shown as surface representation and colored according to its electrostatic potential. The myomesin *L* linker interacting with OL3 is shown as stick representation.

(B) Quantification of endogenous obscurin displaced in neonatal rat cardiomyocytes expressing GFP-fused wild-type My4*L*My5 (*n* = 11) and its T622V (*n* = 10), T622K (*n* = 9), I625T (*n* = 10), V627Y (*n* = 13) variants. Amino acid replacements in the *L* linker inspired by myomesin-2 (T622K and V627Y) and myomesin-3 (I625T) sequences abrogate competition. Error bars are SEM values. ***p ≤ 0.001.

(C) Example of the competitive effect of overexpressed GFP-fused My4*L*My5 (WT) on endogenous obscurin in NRCs. The separate channels for endogenous myomesin, GFP, endogenous obscurin, as well as the combined and ratiometric images with overlaid GFP mask for the outline of the transfected cell are shown. The false-color scale range indicator shows an increased obscurin/myomesin ratio. The scale bar represents 10 μm.

(D–G) Similar to (C) for overexpressed GFP-fused My4*L*My5 T622V (D), T622K (E), I625T (F), and V627Y (G). The scale bar represents 10 μm.

A

Sample	OL3:My4*L*	OL3:My4*L*My5
$MM^{exp/calc}$ (kDa)	20±5/25.2	32±5/36.5
R_g^{exp} (Å)	25.5±2	31.0±2
$R_g^{xt/ai/tC}$ (Å)	28.9/26.1/24.1	NA/31.1/29.8
D_{max}^{exp} (Å)	94.0±5	105.2±5
$D_{max}^{xt/ai/tC}$ (Å)	91.1/96.7/91.3	NA/108.5/108.3
$\chi^{xt/ai/tC}$	1.91/0.86/0.78	NA/1.37/0.71

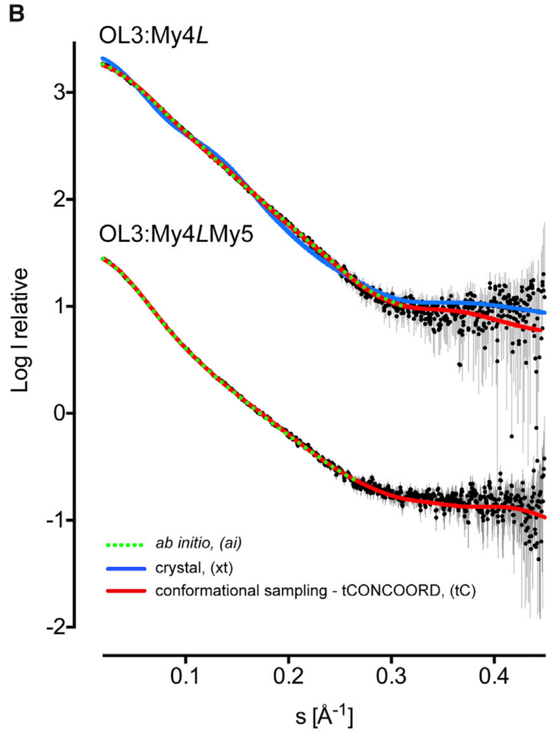

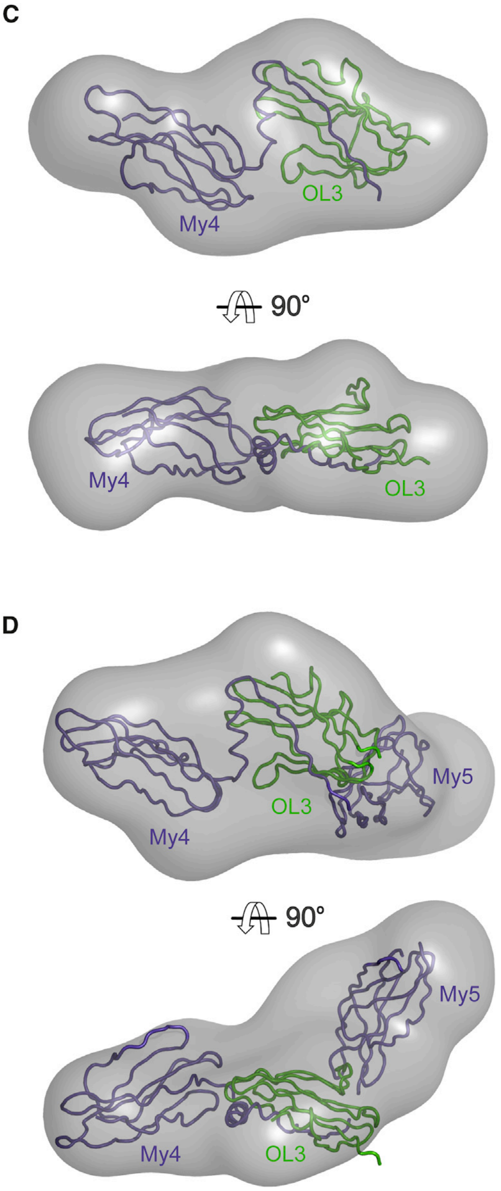

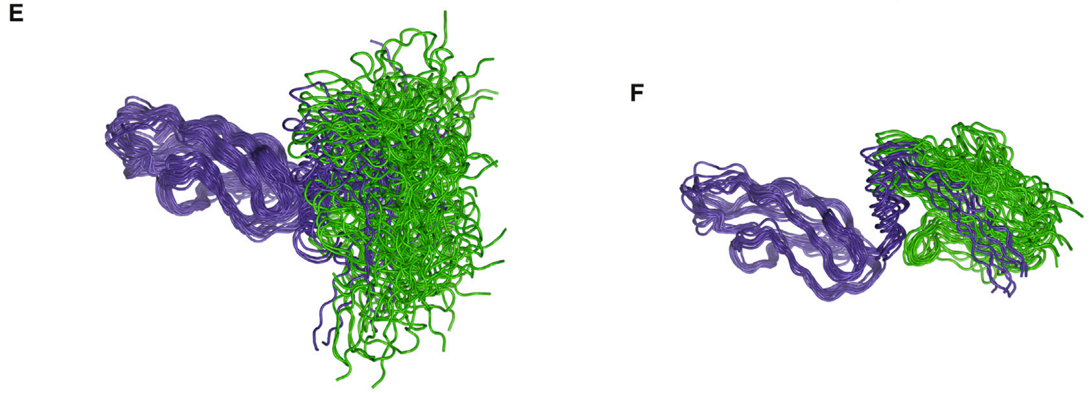

Figure 5. Small-Angle X-Ray Scattering Analysis of the Myomesin:Obscurin-Like-1 Complex

(A) Molecular parameters calculated from SAXS data. MM, R_g, D_{max} are the molecular mass, radius of gyration, and maximum size, respectively. The superscript exp denotes experimental values while xt, *ai*, and tC refer to crystal, ab initio, and *tCONCOORD* fitted models, respectively. MM^{calc} is the theoretical MM computed from the protein sequence. χ is the discrepancy between experimental data and those computed from models.

(legend continued on next page)

CellPress

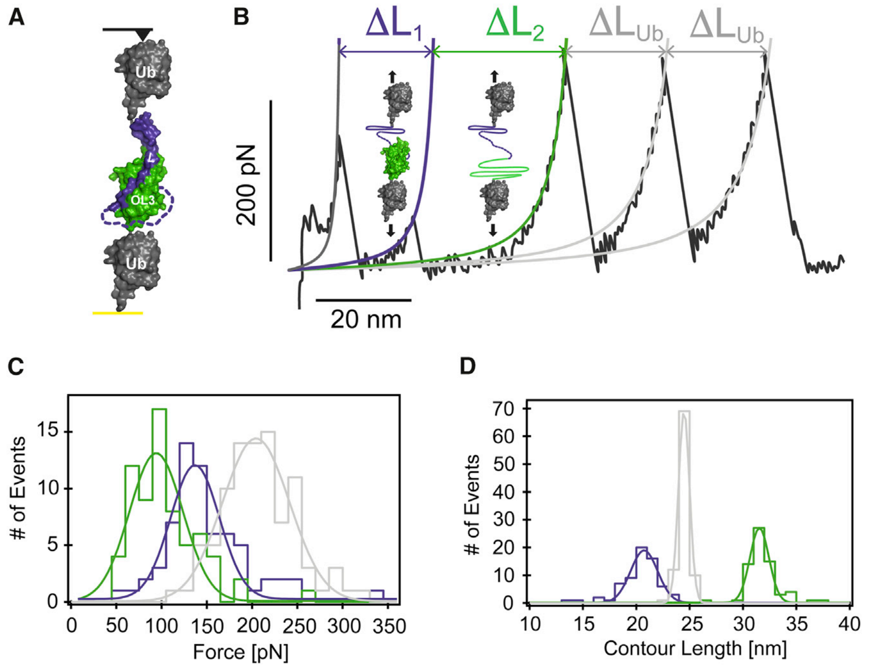

Figure 6. Fold Complementation by the Myomesin *L* Linker Protects the Mechanically Labile OL3 Domain

(A) Schematics of the ubiquitin-*L*-(connector)-OL3-ubiquitin polyprotein used in the single-molecule mechanical experiment. The 43 amino acid flexible connector covalently joining *L* to OL3 is depicted as a dashed line. The color code for *L* and OL3 is as in previous images. Ubiquitin (Ub) molecules bracketing the complex are in gray while the gold substrate and the atomic force microscopy (AFM) cantilever tip are shown in yellow and black, respectively.

(B) Typical force-extension trace for the ubiquitin-*L*-(connector)$_{43}$-OL3-ubiquitin polyprotein exhibiting a saw-tooth pattern of unfolding events that does not follow a hierarchy in the mechanical stability: the first unfolding event occurs at higher forces than the second unfolding event. The unfolding of the ubiquitin modules, occurring at forces of ~200 pN and characterized by an increment of contour length of ΔL_{Ub} ~24.5 nm, ensures the single-molecule nature of the experiment.

(C) Histogram of unfolding forces. The first two events can be readily identified in light of their different mechanical stability and increment in contour length. While the first peak occurs at forces as high as 129.4 ± 27.0 pN (n = 66), the second peak unfolds at the markedly lower force of 86.6 ± 29.1 pN (n = 68).

(D) Histogram of contour length increase: ΔL_1 = 20.2 ± 1.2 nm (n = 66) and ΔL_2 = 31.0 ± 0.9 nm (n = 68). In (C) and (D), colored curves are Gaussian fits.

at the center of the myosin filaments is believed to play a key role as a mechanical safeguard during force-generating cycles and as a signaling hub (Agarkova et al., 2003). Compared with the Z-disk, there is currently limited knowledge of this sarcomeric region. The reason for this is 2-fold. On the one hand, although the identity of some key M-band proteins is well established, new components are steadily emerging, suggesting that a much richer complement resides either stably or transiently at this region. For example, cardiomyopathy associated 5 protein (Cmya5 or myospryn) has been recently shown to bind to M-band titin and calpain-3 (Capn3) protease (Sarparanta et al., 2010). Mutations in Capn3 lead to limb girdle muscle dystrophy (LGDM) type 2A, and secondary Capn3 deficiency occurs in LGMD type 2J. Also, a novel leucine-rich protein named myomasp ([myosin-interacting, M-band-associated stress-responsive protein]/LRRC39) has been detected as an interactor of myosin heavy chain (MYH7), and knockdown of the myomasp/LRRC39 ortholog in zebrafish resulted in severely impaired heart function and cardiomyopathy in vivo (Will et al., 2010). On the other hand, even for known M-band protein components, their complexity is such that their detailed molecular organization is still largely unknown. Thus, advances in our understanding of M-band biology need to address its dynamic proteome, and the mechanical and architectural aspects underpinning its function.

In this work, we explored the myomesin-dependent anchoring of obscurin-like-1 to the M-band and found a mechanism that is new in the sarcomere context. The structure of the obscurin-like-1:myomesin complex reveals that the myomesin *L* linker between its fourth (My4) and fifth (My5) Fn-III domains integrates within the incomplete third Ig domain of obscurin-like-1 (OL3), resulting in a stable protein complex. The mechanism of fold complementation in-*trans* observed for the My4*L*:OL3 complex is somewhat reminiscent of that of subunit-subunit and chaperone-subunit interactions in bacterial pili assembled by the chaperone-usher pathway, whereby the binding partner inserts a β-strand into a partial Ig domain, thus restoring its fold (Remaut et al., 2006). In the case of OL3:My4*L,* this binding mode provides a surprisingly high mechanical stability to the complex (~135 pN), a rupture force significantly higher than that required to unfold OL3 alone (~85 pN) and quantitatively similar to that exhibited by the myomesin C-terminal dimer (~137 pN) (Berkemeier et al., 2011) required for myosin crosslinking. The high force that the complex is able to withstand contrasts with the mechanical lability (~30 pN) measured for the titin:obscurin/obscurin-like-1 complex between M10:OL1/O1 Ig domains

(B) Experimental scattering intensities for OL3:My4*L* (upper curve) and OL3:My4*L*My5 (lower curve) are displayed as dots with error bars. Curves computed from the crystallographic model and the best tC models are shown as solid blue and red lines, respectively, while the curve computed from the ab initio models is shown as a dotted green line.

(C) Cartoon-tube representation of a selection of 20 OL3:My4*L* *tCONCOORD* models (out of >30,000) aligned with respect to their My4 domain. My4*L* is in slate blue and OL3 is in green.

(D) Cartoon-tube representation of the ten OL3:My4*L* *tCONCOORD* models providing the best fit to the SAXS data (0.78 < χ < 0.81).

(E and F) OL3:My4*L* SAXS molecular envelope calculated from the ab initio model with a representative *tCONCOORD* model in two orthogonal orientations; (F) like (E) for OL3:My4*L*My5.

(Pernigo et al., 2010). Such mechanically weaker interaction reflects a completely different structural architecture, based on a parallel β-strand augmentation in an Ig:Ig chevron-shaped zipper module (Pernigo et al., 2010, 2015; Sauer et al., 2010). Interestingly, the low rupture force of the latter interaction is on the order of only about six myosin crossbridges, thus stable anchoring of obscurin-like-1 to the M-band appears to be dependent on its binding to myomesin rather than to titin. Given the high sequence similarity between OL3 and obscurin O3, particularly for the residues involved in the molecular interface with myomesin (Figure 3E), we suggest that the same holds true for obscurin anchoring and that the obscurin:myomesin complex recapitulates OL3:My4*L* in its binding mode. This closely mirrors the behavior of N-terminal Ig domains OL1 and O1 that interact with titin M10 in a mutually exclusive manner using a common interface. However, as for OL1 and O1, where minor, yet significant, structural differences suggest different specificities for putative additional partners (Pernigo et al., 2015), we cannot exclude a similar unanticipated behavior for OL3/O3 as well. Interestingly, both OL3 and O3 are insoluble in bacteria when expressed in isolation, while co-expression in the presence of the myomesin *L* region results in biochemically well-behaved complexes. This suggests a chaperone effect by myomesin, effectively enabling the correct folding of the unconventional augmented O(L)3_A Ig domain. Crucially, removal of the *L* linker from $OL3_A$ results in a semi-folded state with a significantly decreased mechanical stability, requiring only ~85 pN to unfold.

A mechanism of β-strand complementation between linkers or non-structured regions with incomplete Ig domains has also been observed, both in-*cis* and in-*trans*, in Ig domains of the actin crosslinking protein filamin. Filamin A can interact with the cytoplasmic tail of integrin 3 via its Ig-like domain 21 (FLNa21), but FLNa21 can also bind to the linker between FLNa20 and 21 in an intramolecular complex that competes with integrin. Intriguingly, the removal of the *trans*-complemented β-strand from FLNa21 unmasks the binding site for integrin, which, when bound to filamin, engages integrin an inactive state (Heikkinen et al., 2009; Liu et al., 2015). This specific interaction can be opened by mechanical stretch and triggers integrin binding, filamin's partner in mechanosensing (Chen et al., 2009; Seppala et al., 2015). The intermolecular domain *trans*-complementation we observe here for the obscurin(-like-1):myomesin complex might therefore also play a role in mechanosensing, by freeing the O3/OL3 domain for binding to an alternative ligand. As obscurin-like-1 has been linked to ubiquitin-mediated turnover, such a mechanosensing pathway around the obscurin/obscurin-like-1:myomesin complex might feed into the turnover of sarcomere-associated structures (Lange et al., 2012). Myomesin crosslinks myosin filaments and therefore must be exposed at least to some of the shear forces developing transversally to the myosin filament axis, but the extent to which myomesin is directly exposed to mechanical force in vivo remains unknown, not least because the exact orientation with respect to the filament axis can currently be only indirectly inferred, and the geometry of force transmission is therefore unclear. It is also yet unclear in which directionality mechanical forces act on the titin-obscurin, which might be relevant based on recent molecular dynamics simulations (Caldwell et al., 2015). However, it is reasonable to speculate that the extremely stable anchoring of obscurin(-like-1) to myomesin is not only structurally important, but has evolved also as functionally relevant for nanomechanical necessity, supporting the notion that the M-band is a key strain sensor in muscle sarcomeres (Agarkova et al., 2003; Pinotsis et al., 2012; Xiao and Grater, 2014).

The *MYOM* gene family codes for three proteins sharing a similar Ig/Fn-III-rich domain organization. Our OL3:My4*L* structure offers a clear structural basis for the specificity of obscurin(-like-1) binding to the myomesin-1 isoform that was validated by competition assays in the relevant cellular context of NRCs. Interestingly, the OL3:My4*L* complex also reveals interdomain flexibility and the ability to dimerize. The dimeric arrangement observed in the crystal and in solution at high protein concentration opens the possibility that this geometry might reflect the local obscurin(-like-1):myomesin organization in the crowded environment of the sarcomere. The M4/M4′ lines typical of striated muscles define a hexagonal arrangement of myosin filaments in the super-lattices of most vertebrates. Antibody mapping experiments suggested that the N-terminal region of myomesin runs roughly perpendicular to the myosin filament, since My1 and the *L* loop are only 7 nm apart from the M1 line (Obermann et al., 1996). Thus, it is conceivable that myomesin molecules emanating from neighboring myosin filaments of the hexagonal lattice cross over at the level of the *L* linker as seen in the OL3:My4*L* dimer (Figure 7). The intrinsic flexibility of the complex monomer coupled with the presence of the helical spacer at the *L* N-terminus appears perfectly poised for this. This suggestion is compatible with previous M-band models (Lange et al., 2005) but adds a novel geometric constraint. In summary, our work provides a necessary structural and biomechanical reference to establish the geometrical context and mechanical hierarchies in M-band assembly, which will need to be reconciled with more highly resolved in situ information of this protein network and its response to mechanical stress.

EXPERIMENTAL PROCEDURES

Detailed methods used for cloning, protein expression, and protein purification are given in the Supplemental Experimental Procedures.

Crystallization

An initial vapor-diffusion sparse matrix screening performed using the sitting-drop setup with the aid of Mosquito crystallization robot (TTP LabTech) produced hundreds of OL3:My4*L*My5 microcrystals in the presence of 1.1 M ammonium tartrate (pH 7.0) and a 1:2 protein:reservoir ratio. The protein concentration used in the screen was 4.0 mg/mL in storage buffer (20 mM HEPES, 150 mM NaCl, 1 mM DTT [pH 7.5]). A standard pH-precipitant grid optimization allowed us to obtain fewer marginally larger crystals in the presence of 0.8 M ammonium tartrate, 0.1 M sodium acetate (pH 5.5) using a 1:1 protein:reservoir ratio. These crystals, however, proved unsuitable for diffraction experiments. To further improve crystal quality, we employed the random MMS screening approach (D'Arcy et al., 2014). Crystals obtained in the optimization step were harvested and stored in a solution containing 0.9 M ammonium tartrate, 0.1 M sodium acetate (pH 5.5) (hit stock). A new sparse matrix screening was performed using various commercial screens using a hit stock:protein:reservoir ratio of 1:2:1. Few OL3:My4*L*My5 single crystals were finally obtained in the presence of 20% PEG8000, 0.1 M Tris-HCl (pH 8.5), 0.2 M $MgCl_2$ using the protein complex at 3.0 mg/mL. Crystallization of My4L_H is described in the Supplemental Experimental Procedures.

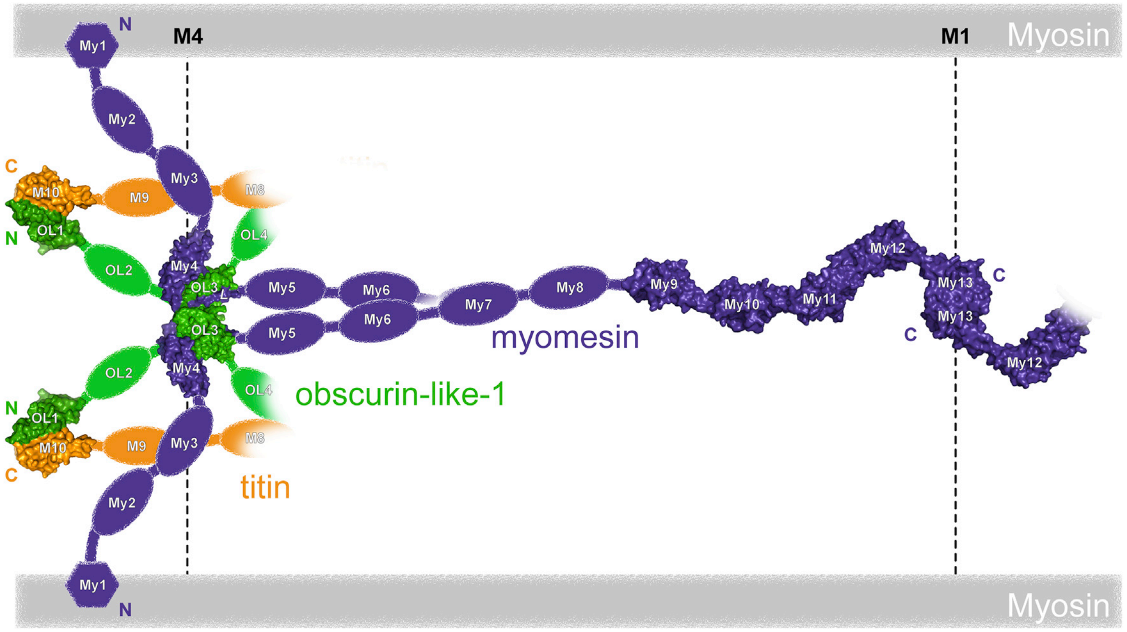

Figure 7. Possible Model for the Organization of the Titin:Obscurin(-like-1):Myomesin Ternary Complex at the M-Band
Experimental complexes are shown as surface representation: OL3:My4*L* dimer (this work), M10:OL1 (Pernigo et al., 2010, 2015; Sauer et al., 2010), My9-M13 (Pinotsis et al., 2008, 2012).

X-Ray Data Collection and Structure Determination

Crystals were cryo-protected by soaking them in their reservoir solution supplemented with 20% glycerol. For OL3:My4*L*My5 a 3.1 Å resolution dataset was collected in space group *C*2 while My4L_H crystallized in the alternative space groups $P6_5$ and *P*2, yielding diffraction data at 2.05 Å and 2.8 Å resolution, respectively. All datasets were collected at Diamond Light Source synchrotron facility (Didcot, Oxfordshire, UK) and processed with the *xia*2 expert system (Winter et al., 2013) using *XDS* (Kabsch, 2010) and *AIMLESS* (Evans and Murshudov, 2013) packages. All X-ray structures were solved by the molecular replacement method with the package *MOLREP* (Vagin and Teplyakov, 2010) and refined using the programs *REFMAC*5 (Murshudov et al., 2011) and *BUSTER* (Bricogne et al., 2011). A summary of data collection and refinement statistics is shown in Table 1. Further details on the crystallographic methods are available in the Supplemental Experimental Procedures.

Cellular Competition Assays in NRCs and Ratiometric Analysis

NRC isolation, culture, transfection, and staining were performed essentially as described previously (Pernigo et al., 2010). Briefly, NRCs were transfected with GFP-tagged transiently expressing constructs (pEGFPC2-, Clontech Laboratories) using Escort III (Sigma-Aldrich). After 48 hr culture to promote protein expression, cells were fixed with 4% paraformaldehyde/PBS, permeabilized with 0.1% Triton X-100/PBS, and then stained with the appropriate antibodies. The antibodies used for the current work were as follows: MyB4, a mouse monoclonal antibody to the myomesin domain My12 (Grove et al., 1984); and Ob5859, a rabbit polyclonal antibody to two consecutive Ig domains in obscurin, Ob58 and Ob59 (Fukuzawa et al., 2005; Young et al., 2001). All fluorescent-conjugated secondary antibodies were purchased from Jackson ImmunoResearch. All images for ratiometry analysis were collected on a Zeiss LSM510 confocal microscope as described previously (Fukuzawa et al., 2008). Image analysis was carried out as described in our previous work (Pernigo et al., 2015). Further details are available in the Supplemental Experimental Procedures.

Small-Angle X-Ray Scattering

Synchrotron SAXS data for OL3:My4*L* and OL3:My4*L*My5 were collected at the BM29 BioSAXS beamline (ESRF, Grenoble) using a Pilatus 1M detector (Dectris) (Pernot et al., 2013). All samples were measured at four concentrations (0.5–4.5 mg/mL in 20 mM HEPES [pH 7.5], 500 mM NaCl, 1 mM DTT buffer) in the range of momentum transfer $0.005 < s < 0.608$ Å^{-1} ($s = 4\pi \sin\theta/\lambda$, where the wavelength λ is 0.9919 Å and 2θ is the scattering angle). All experiments were performed at 18°C using a sample volume of 30 μL loaded into the flowing measurement cell. Individual frames were processed automatically and independently within the *EDNA* framework (Brennich et al., 2016). Merging of separate concentrations and further analysis steps were performed using a combination of tools from the *ATSAS* package (Petoukhov et al., 2012). Initial rigid body modeling of the complex was done with *CORAL* (Petoukhov et al., 2012) and domain dynamics of the protein complexes was further explored by generating conformational ensembles using the *tCONCOORD* (Seeliger et al., 2007) method. Further details are available in the Supplemental Experimental Procedures.

Single-Molecule Mechanical Experiments by Atomic Force Microscopy

cDNA was commercially synthesized (Genscript), which allowed the expression of a polyprotein in which the myomesin linker *L* and the obscurin-like-1 OL3 domain are connected by a flexible 43-amino-acid-long connector sandwiched between two ubiquitin (Ub) domains (Ub-*L*-connector-OL3-Ub). The synthetic gene was inserted into a pQE80L vector (QIAGEN) using standard molecular biology techniques. A PCR-based approach also allowed the extension of the connector length to 64 amino acids. Single proteins were picked up from the surface and pulled at a constant velocity of 400 nm s^{-1} (del Rio et al., 2009; Garcia-Manyes et al., 2012; Perez-Jimenez et al., 2006). Further details are available in the Supplemental Experimental Procedures.

ACCESSION NUMBERS

Atomic coordinates for the X-ray structures presented in this article have been deposited with the PDB under accession codes PDB: 5FM4, 5FM5, 5FM8.

SUPPLEMENTAL INFORMATION

Supplemental Information includes Supplemental Experimental Procedures, eight figures, and one movie and can be found with this article online at http://dx.doi.org/10.1016/j.str.2016.11.015.

AUTHOR CONTRIBUTIONS

R.A.S. and M.G. designed the research. S.P. purified proteins and performed the crystallographic and SAXS work. A.R. analyzed SAXS data. A.P. generated models for SAXS analysis. A.F. and M.H. carried out the cellular competition assay and its analysis. A.E.M.B. and S.G.-M. performed and analyzed the AFM data. R.A.S. wrote the original draft. R.A.S., S.G.-M., and M.G. supervised the research and wrote the paper. All authors reviewed and contributed to the manuscript.

ACKNOWLEDGMENTS

We thank the Diamond Light Source and the European Synchrotron Radiation Laboratory for access to MX and SAXS beamlines, respectively. This work was supported by a British Heart Foundation grant (PG/10/67/28527) awarded to R.A.S. and M.G. as well as MRC grant MR/J010456/1 to M.G. and a British Heart Foundation grant (PG/13/50/30426) and EPSRC Fellowship (K00641X/1) to S.G.-M.

Received: July 22, 2016
Revised: September 16, 2016
Accepted: November 18, 2016
Published: December 15, 2016

REFERENCES

Agarkova, I., Ehler, E., Lange, S., Schoenauer, R., and Perriard, J.C. (2003). M-band: a safeguard for sarcomere stability? J. Muscle Res. Cell Motil. *24*, 191–203.

Arimura, T., Matsumoto, Y., Okazaki, O., Hayashi, T., Takahashi, M., Inagaki, N., Hinohara, K., Ashizawa, N., Yano, K., and Kimura, A. (2007). Structural analysis of obscurin gene in hypertrophic cardiomyopathy. Biochem. Biophys. Res. Commun. *362*, 281–287.

Bagnato, P., Barone, V., Giacomello, E., Rossi, D., and Sorrentino, V. (2003). Binding of an ankyrin-1 isoform to obscurin suggests a molecular link between the sarcoplasmic reticulum and myofibrils in striated muscles. J. Cell Biol. *160*, 245–253.

Benian, G.M., Tinley, T.L., Tang, X., and Borodovsky, M. (1996). The *Caenorhabditis elegans* gene unc-89, required fpr muscle M-line assembly, encodes a giant modular protein composed of Ig and signal transduction domains. J. Cell Biol. *132*, 835–848.

Berkemeier, F., Bertz, M., Xiao, S., Pinotsis, N., Wilmanns, M., Grater, F., and Rief, M. (2011). Fast-folding alpha-helices as reversible strain absorbers in the muscle protein myomesin. Proc. Natl. Acad. Sci. USA *108*, 14139–14144.

Brennich, M.E., Kieffer, J., Bonamis, G., De Maria Antolinos, A., Hutin, S., Pernot, P., and Round, A. (2016). Online data analysis at the ESRF bioSAXS beamline. BM29. J. Appl. Cryst. *49*, 203–212.

Bricogne, G., Blanc, E., Brandl, M., Flensburg, C., Keller, P., Paciorek, W., Roversi, P., Sharff, A., Smart, O.S., Vonrhein, C., et al. (2011). BUSTER Version 2.10.0 (Global Phasing).

Caldwell, T.A., Sumner, I., and Wright, N.T. (2015). Mechanical dissociation of the M-band titin/obscurin complex is directionally dependent. FEBS Lett. *589*, 1735–1739.

Carmignac, V., Salih, M.A., Quijano-Roy, S., Marchand, S., Al Rayess, M.M., Mukhtar, M.M., Urtizberea, J.A., Labeit, S., Guicheney, P., Leturcq, F., et al. (2007). C-terminal titin deletions cause a novel early-onset myopathy with fatal cardiomyopathy. Ann. Neurol. *61*, 340–351.

Carrion-Vazquez, M., Li, H., Lu, H., Marszalek, P.E., Oberhauser, A.F., and Fernandez, J.M. (2003). The mechanical stability of ubiquitin is linkage dependent. Nat. Struct. Biol. *10*, 738–743.

Chen, H.S., Kolahi, K.S., and Mofrad, M.R. (2009). Phosphorylation facilitates the integrin binding of filamin under force. Biophys. J. *97*, 3095–3104.

D'Arcy, A., Bergfors, T., Cowan-Jacob, S.W., and Marsh, M. (2014). Microseed matrix screening for optimization in protein crystallization: what have we learned? Acta Crystallogr. F Struct. Biol. Commun. *70*, 1117–1126.

del Rio, A., Perez-Jimenez, R., Liu, R., Roca-Cusachs, P., Fernandez, J.M., and Sheetz, M.P. (2009). Stretching single talin rod molecules activates vinculin binding. Science *323*, 638–641.

Evans, P.R., and Murshudov, G.N. (2013). How good are my data and what is the resolution? Acta Crystallogr. D Biol. Crystallogr. *69*, 1204–1214.

Fukuzawa, A., Idowu, S., and Gautel, M. (2005). Complete human gene structure of obscurin: implications for isoform generation by differential splicing. J. Muscle Res. Cell Motil. *26*, 427–434.

Fukuzawa, A., Lange, S., Holt, M., Vihola, A., Carmignac, V., Ferreiro, A., Udd, B., and Gautel, M. (2008). Interactions with titin and myomesin target obscurin and obscurin-like 1 to the M-band: implications for hereditary myopathies. J. Cell Sci. *121*, 1841–1851.

Garcia-Manyes, S., Badilla, C.L., Alegre-Cebollada, J., Javadi, Y., and Fernandez, J.M. (2012). Spontaneous dimerization of titin protein Z1Z2 domains induces strong nanomechanical anchoring. J. Biol. Chem. *287*, 20240–20247.

Gautel, M., and Djinovic-Carugo, K. (2016). The sarcomeric cytoskeleton: from molecules to motion. J. Exp. Biol. *219*, 135–145.

Grove, B.K., Kurer, V., Lehner, C., Doetschman, T.C., Perriard, J.C., and Eppenberger, H.M. (1984). A new 185,000-dalton skeletal muscle protein detected by monoclonal antibodies. J. Cell Biol. *98*, 518–524.

Hanson, D., Murray, P.G., Sud, A., Temtamy, S.A., Aglan, M., Superti-Furga, A., Holder, S.E., Urquhart, J., Hilton, E., Manson, F.D., et al. (2009). The primordial growth disorder 3-M syndrome connects ubiquitination to the cytoskeletal adaptor OBSL1. Am. J. Hum. Genet. *84*, 801–806.

Harpaz, Y., and Chothia, C. (1994). Many of the immunoglobulin superfamily domains in cell adhesion molecules and surface receptors belong to a new structural set which is close to that containing variable domains. J. Mol. Biol. *238*, 528–539.

Heikkinen, O.K., Ruskamo, S., Konarev, P.V., Svergun, D.I., Iivanainen, T., Heikkinen, S.M., Permi, P., Koskela, H., Kilpelainen, I., and Ylanne, J. (2009). Atomic structures of two novel immunoglobulin-like domain pairs in the actin cross-linking protein filamin. J. Biol. Chem. *284*, 25450–25458.

Horowits, R., Kempner, E.S., Bisher, M.E., and Podolsky, R.J. (1986). A physiological role for titin and nebulin in skeletal muscle. Nature *323*, 160–164.

Hu, L.Y., and Kontrogianni-Konstantopoulos, A. (2013). The kinase domains of obscurin interact with intercellular adhesion proteins. FASEB J. *27*, 2001–2012.

Huber, C., Fradin, M., Edouard, T., Le Merrer, M., Alanay, Y., Da Silva, D.B., David, A., Hamamy, H., van Hest, L., Lund, A.M., et al. (2009). OBSL1 mutations in 3-M syndrome are associated with a modulation of IGFBP2 and IGFBP5 expression levels. Hum. Mutat. *31*, 20–26.

Kabsch, W. (2010). Xds. Acta Crystallogr. D Biol. Crystallogr. *66*, 125–132.

Katzemich, A., Kreiskother, N., Alexandrovich, A., Elliott, C., Schock, F., Leonard, K., Sparrow, J., and Bullard, B. (2012). The function of the M-line protein obscurin in controlling the symmetry of the sarcomere in the flight muscle of *Drosophila*. J. Cell Sci. *125*, 3367–3379.

Katzemich, A., West, R.J., Fukuzawa, A., Sweeney, S.T., Gautel, M., Sparrow, J., and Bullard, B. (2015). Binding partners of the kinase domains in Drosophila obscurin and their effect on the structure of the flight muscle. J. Cell Sci. *128*, 3386–3397.

Kontrogianni-Konstantopoulos, A., Jones, E.M., Van Rossum, D.B., and Bloch, R.J. (2003). Obscurin is a ligand for small ankyrin 1 in skeletal muscle. Mol. Biol. Cell *14*, 1138–1148.

Krissinel, E., and Henrick, K. (2007). Inference of macromolecular assemblies from crystalline state. J. Mol. Biol. *372*, 774–797.

Lange, S., Agarkova, I., Perriard, J.C., and Ehler, E. (2005). The sarcomeric M-band during development and in disease. J. Muscle Res. Cell Motil. *26*, 375–379.

Lange, S., Perera, S., Teh, P., and Chen, J. (2012). Obscurin and KCTD6 regulate cullin-dependent small ankyrin-1 (sAnk1.5) protein turnover. Mol. Biol. Cell *23*, 2490–2504.

Li, H., and Fernandez, J.M. (2003). Mechanical design of the first proximal Ig domain of human cardiac titin revealed by single molecule force spectroscopy. J. Mol. Biol. *334*, 75–86.

Liu, J., Das, M., Yang, J., Ithychanda, S.S., Yakubenko, V.P., Plow, E.F., and Qin, J. (2015). Structural mechanism of integrin inactivation by filamin. Nat. Struct. Mol. Biol. *22*, 383–389.

Marston, S., Montgiraud, C., Munster, A.B., Copeland, O., Choi, O., Dos Remedios, C., Messer, A.E., Ehler, E., and Knoll, R. (2015). OBSCN mutations associated with dilated cardiomyopathy and haploinsufficiency. PLoS One *10*, e0138568.

Murshudov, G.N., Skubak, P., Lebedev, A.A., Pannu, N.S., Steiner, R.A., Nicholls, R.A., Winn, M.D., Long, F., and Vagin, A.A. (2011). REFMAC5 for the refinement of macromolecular crystal structures. Acta Crystallogr. D Biol. Crystallogr. *67*, 355–367.

Obermann, W.M., Gautel, M., Steiner, F., van der Ven, P.F., Weber, K., and Furst, D.O. (1996). The structure of the sarcomeric M band: localization of defined domains of myomesin, M-protein, and the 250-kD carboxy-terminal region of titin by immunoelectron microscopy. J. Cell Biol. *134*, 1441–1453.

Otey, C.A., Dixon, R., Stack, C., and Goicoechea, S.M. (2009). Cytoplasmic Ig-domain proteins: cytoskeletal regulators with a role in human disease. Cell. Motil. Cytoskeleton *66*, 618–634.

Peng, Q., and Li, H. (2009). Domain insertion effectively regulates the mechanical unfolding hierarchy of elastomeric proteins: toward engineering multifunctional elastomeric proteins. J. Am. Chem. Soc. *131*, 14050–14056.

Perez-Jimenez, R., Garcia-Manyes, S., Ainavarapu, S.R., and Fernandez, J.M. (2006). Mechanical unfolding pathways of the enhanced yellow fluorescent protein revealed by single molecule force spectroscopy. J. Biol. Chem. *281*, 40010–40014.

Pernigo, S., Fukuzawa, A., Bertz, M., Holt, M., Rief, M., Steiner, R.A., and Gautel, M. (2010). Structural insight into M-band assembly and mechanics from the titin-obscurin-like-1 complex. Proc. Natl. Acad. Sci. USA *107*, 2908–2913.

Pernigo, S., Fukuzawa, A., Pandini, A., Holt, M., Kleinjung, J., Gautel, M., and Steiner, R.A. (2015). The crystal structure of the human titin:obscurin complex reveals a conserved yet specific muscle M-band zipper module. J. Mol. Biol. *427*, 718–736.

Pernot, P., Round, A., Barrett, R., De Maria Antolinos, A., Gobbo, A., Gordon, E., Huet, J., Kieffer, J., Lentini, M., Mattenet, M., et al. (2013). Upgraded ESRF BM29 beamline for SAXS on macromolecules in solution. Journal of Synchrotron Radiation *20* (Pt 4), 660–664.

Petoukhov, M.V., Franke, D., Shkumatov, A.V., Tria, G., Kikhney, A.G., Gajda, M., Gorba, C., Mertens, H.D., Konarev, P.V., and Svergun, D.I. (2012). New developments in the program package for small-angle scattering data analysis. J. Appl. Crystallogr. *45*, 342–350.

Pinotsis, N., Lange, S., Perriard, J.C., Svergun, D.I., and Wilmanns, M. (2008). Molecular basis of the C-terminal tail-to-tail assembly of the sarcomeric filament protein myomesin. EMBO J. *27*, 253–264.

Pinotsis, N., Chatziefthimiou, S.D., Berkemeier, F., Beuron, F., Mavridis, I.M., Konarev, P.V., Svergun, D.I., Morris, E., Rief, M., and Wilmanns, M. (2012). Superhelical architecture of the myosin filament-linking protein myomesin with unusual elastic properties. PLoS Biol. *10*, e1001261.

Pollazzon, M., Suominen, T., Penttila, S., Malandrini, A., Carluccio, M.A., Mondelli, M., Marozza, A., Federico, A., Renieri, A., Hackman, P., et al. (2009). The first Italian family with tibial muscular dystrophy caused by a novel titin mutation. J. Neurol. *257*, 575–579.

Qadota, H., Blangy, A., Xiong, G., and Benian, G.M. (2008). The DH-PH region of the giant protein UNC-89 activates RHO-1 GTPase in Caenorhabditis elegans body wall muscle. J. Mol. Biol. *383*, 747–752.

Raeker, M.O., and Russell, M.W. (2011). Obscurin depletion impairs organization of skeletal muscle in developing zebrafish embryos. J. Biomed. Biotechnol. *2011*, 479135.

Randazzo, D., Giacomello, E., Lorenzini, S., Rossi, D., Pierantozzi, E., Blaauw, B., Reggiani, C., Lange, S., Peter, A.K., Chen, J., et al. (2013). Obscurin is required for ankyrinB-dependent dystrophin localization and sarcolemma integrity. J. Cell Biol. *200*, 523–536.

Remaut, H., Rose, R.J., Hannan, T.J., Hultgren, S.J., Radford, S.E., Ashcroft, A.E., and Waksman, G. (2006). Donor-strand exchange in chaperone-assisted pilus assembly proceeds through a concerted beta strand displacement mechanism. Mol. Cell *22*, 831–842.

Sarparanta, J., Blandin, G., Charton, K., Vihola, A., Marchand, S., Milic, A., Hackman, P., Ehler, E., Richard, I., and Udd, B. (2010). Interactions with M-band titin and calpain 3 link myospryn (CMYA5) to tibial and limb-girdle muscular dystrophies. J. Biol. Chem. *285*, 30304–30315.

Sauer, F., Vahokoski, J., Song, Y.H., and Wilmanns, M. (2010). Molecular basis of the head-to-tail assembly of giant muscle proteins obscurin-like 1 and titin. EMBO Rep. *11*, 534–540.

Schoenauer, R., Lange, S., Hirschy, A., Ehler, E., Perriard, J.C., and Agarkova, I. (2008). Myomesin 3, a novel structural component of the M-band in striated muscle. J. Mol. Biol. *376*, 338–351.

Seeliger, D., Haas, J., and de Groot, B.L. (2007). Geometry-based sampling of conformational transitions in proteins. Structure *15*, 1482–1492.

Seppala, J., Tossavainen, H., Rodic, N., Permi, P., Pentikainen, U., and Ylanne, J. (2015). Flexible structure of peptide-bound filamin a mechanosensor domain pair 20-21. PLoS One *10*, e0136969.

Small, T.M., Gernert, K.M., Flaherty, D.B., Mercer, K.B., Borodovsky, M., and Benian, G.M. (2004). Three new isoforms of *Caenorhabditis elegans* UNC-89 containing MLCK-like protein kinase domains. J. Mol. Biol. *342*, 91–108.

Spooner, P.M., Bonner, J., Maricq, A.V., Benian, G.M., and Norman, K.R. (2012). Large isoforms of UNC-89 (obscurin) are required for muscle cell architecture and optimal calcium release in *Caenorhabditis elegans*. PLoS One *7*, e40182.

Vagin, A., and Teplyakov, A. (2010). Molecular replacement with MOLREP. Acta Crystallogr. D Biol. Crystallogr. *66*, 22–25.

Waterston, R.H., Thomson, J.N., and Brenner, S. (1980). Mutants with altered muscle structure of *Caenorhabditis elegans*. Dev. Biol. *77*, 271–302.

Will, R.D., Eden, M., Just, S., Hansen, A., Eder, A., Frank, D., Kuhn, C., Seeger, T.S., Oehl, U., Wiemann, S., et al. (2010). Myomasp/LRRC39, a heart- and muscle-specific protein, is a novel component of the sarcomeric M-band and is involved in stretch sensing. Circ. Res. *107*, 1253–1264.

Wilson, K.J., Qadota, H., Mains, P.E., and Benian, G.M. (2012). UNC-89 (obscurin) binds to MEL-26, a BTB-domain protein, and affects the function of MEI-1 (katanin) in striated muscle of *Caenorhabditis elegans*. Mol. Biol. Cell *23*, 2623–2634.

Winter, G., Lobley, C.M., and Prince, S.M. (2013). Decision making in xia2. Acta Crystallogr. D Biol. Crystallogr. *69*, 1260–1273.

Xiao, S., and Grater, F. (2014). Molecular basis of the mechanical hierarchy in myomesin dimers for sarcomere integrity. Biophys. J. *107*, 965–973.

Young, P., Ehler, E., and Gautel, M. (2001). Obscurin, a giant sarcomeric Rho guanine nucleotide exchange factor protein involved in sarcomere assembly. J. Cell Biol. *154*, 123–136.

Structure, Volume *25*

Supplemental Information

Binding of Myomesin to Obscurin-Like-1 at the Muscle M-Band Provides a Strategy for Isoform-Specific Mechanical Protection

Stefano Pernigo, Atsushi Fukuzawa, Amy E.M. Beedle, Mark Holt, Adam Round, Alessandro Pandini, Sergi Garcia-Manyes, Mathias Gautel, and Roberto A. Steiner

Supplemental information for

Binding of myomesin to obscurin-like-1 at the muscle M-band provides a strategy for isoform-specific mechanical protection

Stefano Pernigo[1], Atsushi Fukuzawa[1,2], Amy E. M. Beedle[1,3], Mark Holt[1,2], Adam Round[4], Alessandro Pandini[1,5], Sergi Garcia-Manyes[1,3,*], Mathias Gautel[1,2,*], Roberto A. Steiner[1,*]

[1]Randall Division of Cell and Molecular Biophysics, King's College London, London, SE1 1UL, UK

[2]Cardiovascular Division, King's College London BHF Centre of Research Excellence, London, SE1 1UL, UK

[3]Department of Physics, King’s College London, London, WC2R 2LS, UK

[4]European Molecular Biology Laboratory, Grenoble Outstation, 38042 Grenoble, France

[5]Department of Computer Science and Synthetic Biology Theme, Brunel University London, UB8 3PH, UK

Contact

Correspondence should be addressed to

Email: roberto.steiner@kcl.ac.uk

Email: mathias.gautel@kcl.ac.uk

Email: sergi.garcia-manyes@kcl.ac.uk

Lead author

Email: roberto.steiner@kcl.ac.uk

SI Materials and Methods

Cloning

The sequence for the third Ig domain of either human obscurin (residues 238-334, O3) or human obscurin-like 1 (residues 251-339, OL3) was subcloned between the EcoRI/SalI sites of the first expression cassette of an in-house generated pGEX-6P-2rbs vector (a kind gift of the Musacchio Lab, MPI, Dortmund). The pGEX-6P-2rbs vector is based on the pGEX-6P vector (GE Healthcare) but contains two expression cassettes separated by a ribosome binding site. Proteins expressed from the first cassette are C-terminal fusions to GST. The sequence for human myomesin encompassing the fourth and fifth Fn-III domains (residues 510-739, My4*L*My5) was cloned between the BglII/XhoI restriction sites of the second expression cassette. Constructs expressing a deletion version of My4*L*My5 in which the fifth Fn-III domain is removed (residues 510-635, My4*L*) were generated using the QuikChange kit (Stratagene) by replacing the triplet coding for residue I636 with a stop codon. Additionally, cDNA corresponding to myomesin residues 510-618 (My4L_H) was cloned in an in-house modified pET9 vector. Downstream the His_6-tag, this vector encodes for a tobacco etch virus (TEV) protease recognition sequence. All constructs were verified by sequencing.

Protein expression and purification

All vectors were independently transformed into the BL21 (DE3) *E. coli* strain to allow protein expression using standard methods. GST-obscurin(-like-1):myomesin complexes were purified by incubating the soluble fraction with Glutathione Superflow beads (Generon) previously equilibrated in lysis buffer for 3h at 4° C. After incubation the beads were extensively washed and resuspended in lysis buffer.

The GST tag was removed by O/N incubation of the beads suspension with PreScission protease (GE healthcare) at 4° C with gentle shaking. Untagged complexes were separated from the resin–bound protein material by centrifugation at 400g for 15 minutes. All complexes were further purified by ion-exchange chromatography (IEC) and extensively dialyzed at 4°C against 50 mM Tris/HCl, 15 mM NaCl, 1.0 mM DTT, pH 8.5 and 50 mM MES, 15 mM NaCl, 1.0 mM DTT, pH 6.0, respectively. The O3:My4*L*(My5): and OL3:My4*L*(My5) complexes were loaded onto ResourceQ and ResourceS columns (GE healthcare), respectively, and eluted using a NaCl linear gradient. Fractions containing the complexes were pooled, concentrated and further purified by size-exclusion chromatography (SEC) on a 16⁄60 HiLoad Superdex 75 column (GE Healthcare) equilibrated with 20 mM Hepes, 50 mM NaCl, 1 mM DTT, pH 7.5. His_6-My4L_H was purified by immobilised metal affinity purification (IMAC) followed by His_6-tag cleavage using TEV protease. Untagged My4L_H was separated from the uncleaved material by a second round of IMAC. Flow-through fractions containing My4α were pooled, concentrated and further purified by (SEC) on a 16⁄60 HiLoad Superdex 75 column (GE Healthcare) equilibrated with 20 mM HEPES, 150 mM NaCl, 1 mM DTT, pH 7.5.

Crystallisation of My4L_H

Crystallization of My4L_H was accomplished using the vapor-diffusion method by concentrating the protein at 20 mg/ml. Optimization of hits obtained using various commercial screens produced diffracting crystals belonging to space group $P2_1$ and $P6_5$ in 25% (w/v) PEG 3350, 0.1 M BIS/Tris pH 5.5 and 21% (w/v) PEG 2000 MME, 0.1 M Tris-HCl pH 8.8, 0.010 M $NiCl_2$, respectively.

X-ray data collection and structure determination

For the OL3:My4*L*My5 complex Matthews' coefficient calculations (Matthews, 1968) suggested a V_M of 2.87 $Å^3Da^{-1}$ as the most probable value. This implied the presence of two OL3:My4*L*My5 heterodimers in the a.u. with a solvent content of 57.2%. Using the Fn-III domain of 2NZI (Mrosek et al., 2007) as template, molecular replacement (MR) attempts performed using the program *MOLREP* (Vagin and Teplyakov, 2010) identified two Fn-III domains related by a 2-fold symmetry axis along the z axis. Further attempts at locating two additional Fn-III domains by MR were unsuccessful. We then decided to turn our attention to the OL3 domain. Using a truncated poly-Ala version of the OL1 Ig domain (PDB code 2WP3 (Pernigo et al., 2010), a new MR search positioned two Ig domains obeying the same dyad symmetry of the previously positioned Fn-III domains. Model building was carried out manually using the program *COOT* (Emsley et al., 2010) and structure refinement was performed using the programs *REFMAC*5 (Murshudov et al., 2011) and *BUSTER* (Bricogne et al., 2011). Electron density maps calculated during the refinement process failed to reveal information on the missing Fn-III domains. The final deposited structure (PDB code, 5FM5) consists of a pair of OL3:My4*L* heterodimers assembled as a dimer in the crystal. The structure of My4L_H was solved in space groups $P6_5$ and $P2_1$ by MR starting from the relevant portion of 5FM5 as template. Final models have been deposited with the PDB with accession codes 5FM8 and 5FM4, respectively. A summary of refinement statistics is shown in (Table 1).

Cellular competition assays in NRCs and ratiometric analysis

Briefly, to see the competitive effect of overexpressed GFP-My4*L*My5 and their variants on endogenous obscurin, we imported 16-bit-per-channel RGB files into Mathematica 10 (Wolfram Research) and translated them to ensure correct alignment

of the myomesin and obscurin channels. Background subtraction was performed on these channels by subtracting a 50-pixel radius Gaussian filtered version of that channel. The arctangent of the obscurin and myomesin intensities was then calculated on a pixel-by-pixel basis to give a ratio image of obscurin to myomesin that was linearly scaled between 0.0 and 1.0. A value of 0.0 corresponded to no obscurin in the presence of myomesin, while a value of 1.0 corresponded to obscurin in the absence of myomesin. A value of 0.5 corresponded to equal intensities in both channels. The GFP channel was binarised to create a segmented image corresponding to GFP-positive and GFP-negative regions. The ratio image was multiplied by this mask and separately with an inverted version of this mask to give two ratio images that corresponded to the GFP-positive and GFP-negative regions of the image as two separate images. A histogram for each image was then determined and normalized to a total of 1 for each histogram. Plotting these against each other thus allowed comparison in shifts in ratio. A left shift (towards zero) indicated a reduction of obscurin in the GFP-positive ratio image. The difference between the two curves in this direction was calculated and the data presented represent one minus the total integrated difference between these two. Thus, a value of 1.0 represents total competition of endogenous obscurin by the GFP fusion, while a value of 0.0 represented absence of competition. Data in Figure 5B are reported normalized to the obscurin competition level of GFP-My4LMy5wt (0.504±0.092, mean±s.e.m., n=11).

Small angle X-ray scattering (SAXS)

All experiments were performed at 18 °C using a sample volume of 30 μl loaded into the flowing measurement cell. Individual frames were processed automatically and independently within the *EDNA* framework, yielding individual radially averaged curves of normalized intensity. Matched buffer measurements taken before and after

every sample were averaged and used for background subtraction. Merging of separate concentrations and further analysis steps were performed using a combination of tools from the *ATSAS* package (Petoukhov et al., 2012). Standard procedures were applied for the computation of the forward scattering $I(0)$, radius of gyration R_g, and maximum dimension D_{max}. The molecular mass of the solutes was evaluated by comparison with reference solutions of BSA. For *ab initio* envelope reconstruction a total of 40 models were calculated for each construct using *DAMMIF* (Franke and Svergun, 2009), and then averaged, aligned and compared using *DAMAVER* (Volkov and Svergun, 2003), the output of which was used in the final refinement to the scattering data with *DAMMIN* (Svergun, 1999). Initial rigid body modelling of the complex was done with *CORAL* (Petoukhov et al., 2012) and domain dynamics of the protein complexes was further explored by generating conformational ensembles using the *tCONCOORD* (Seeliger et al., 2007) method. A total of 32768 structures were generated for each complex with this method. Theoretical scattering and comparison to the experimental data was done with *PepsiSAXS*.

Single-molecule mechanical experiments by atomic force microscopy (AFM)

An N-terminal His_6 tag is also present to facilitate protein purification and two C-terminal Cys residues are available at the protein C-terminus to allow protein attachment to the atomic force microscope. The His_6-Ub-*L*-connector-OL3-Ub-CC polyproteins were purified using a combination of IMAC, IEC and SEC techniques. Constant velocity AFM experiments were conducted at room temperature using both a home-made set-up (Schlierf et al., 2004) and a commercial Luigs and Neumann force spectrometer (Popa et al., 2013). In all cases, the sample was prepared by depositing 1-10 μL of protein in PBS solution (at a concentration of 1-10 mg ml^{-1})

onto a freshly evaporated gold slide. Each cantilever (Si_3N_4 Bruker MLCT-AUHW) was individually calibrated using the equipartition theorem, giving rise to a typical spring constant of ~12 pN nm^{-1}.

Figure S1.

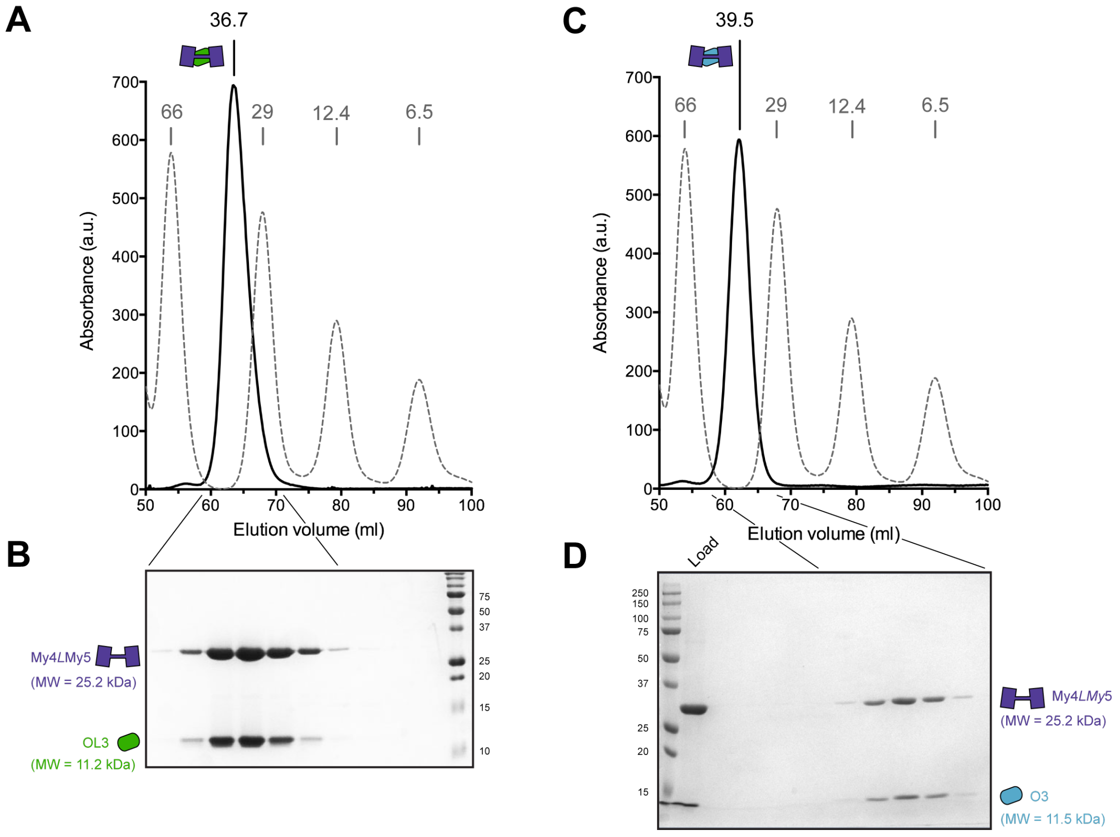

Figure S1 (related to Figure 1B, inset). OL3:My4*L*My5 and O3:My4*L*My5 complexes. (A,C) During purification OL3:My4*L*My5 (A) and O3:My4*L*My5 (C) (black continuous traces) elute as 1:1 stoichiometric complexes with an apparent molecular weight (MW) of 36.7 kDa and 39.5, respectively, from a Superdex 75 16/600 size-exclusion column. The calculated MW is 36.4 kDa and 36.8 kDa for OL3:My4*L*My5 and O3:My4*L*My5, respectively. The elution profile of calibration MW markers is shown with a broken grey line together with their MW in kDa; (B, D) Fractions of the OL3:My4*L*My5 (B) and O3:My4*L*My5 (D) peaks were analyzed by SDS–PAGE.

Figure S2.

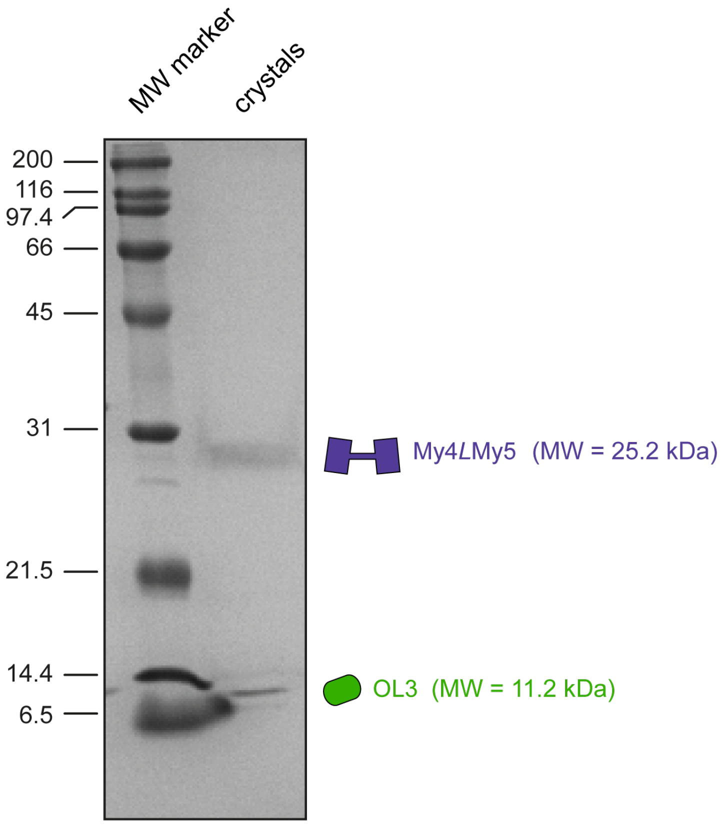

Figure S2. The OL3:My4*L*My5 complex is not proteolyzed in the crystal (related to Figure 1B, inset). OL3:My4*L*My5 crystals were extensively washed, dissolved in buffer, and analyzed by SDS-PAGE. Both My4*L*My5 and OL3 domains migrate according to their expected molecular weight (MW) indicating that proteolysis did not occur during the crystallization process. The lack of electron density for the My5 domain in crystallographic maps is likely due to flexibility of the hinge region connecting My4*L* to My5. The MW of protein markers is given on the left-hand.

Figure S3.

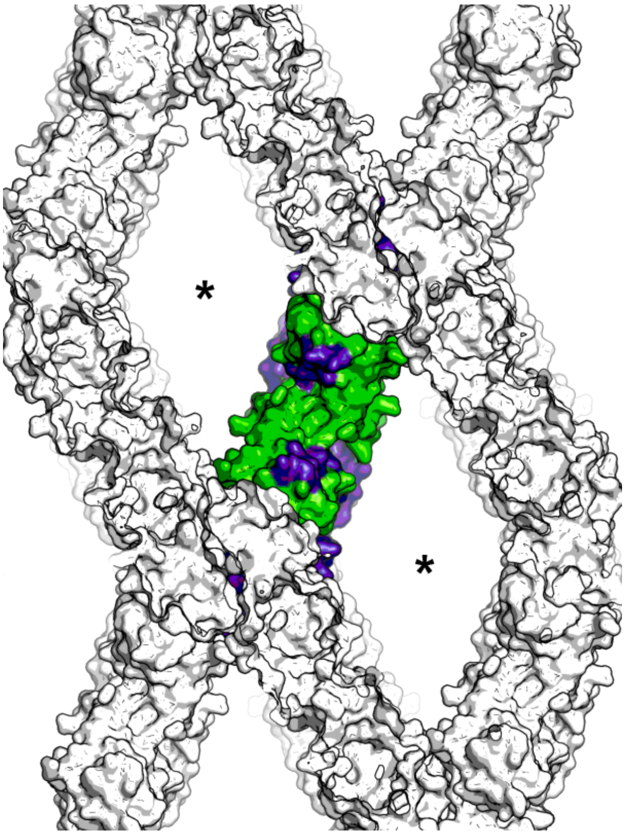

Figure S3. OL3:My4*L*My5 crystal packing (related to Figure 2A). A sliced view of the molecular arrangement in the crystal represented along the 2-fold $(OL3:My4L)_2$ axis. Large channels, highlighted by asterisks, can accommodate the My5 domain. A single $(OL3:My4L)_2$ complex is highlighted with My4*L* shown in slate blue and OL3 in green. Symmetry related molecules are represented in white.

Figure S4.

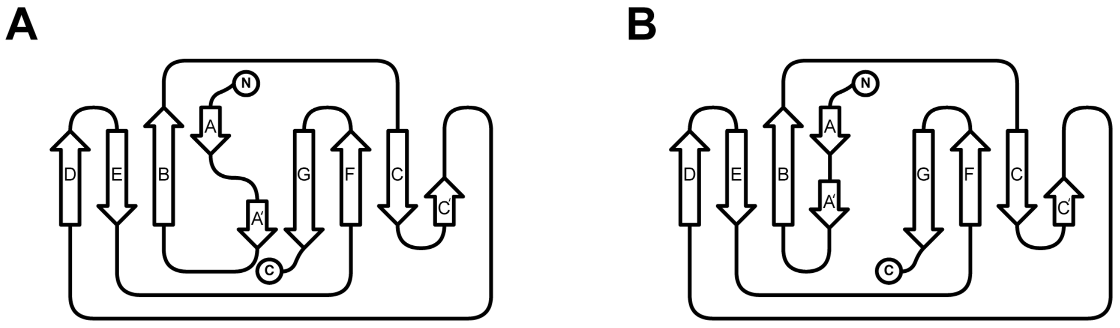

Figure S4. Intermediate-set (I-set) Ig topologies (related to Figure 3C, inset). Topology diagrams of (A) I-set and (B) I*-set Ig-fold domains. The I*-set exhibits a relocation of the A' strand (Pernigo et al., 2015). Strands are labelled according to the classical Ig-fold nomenclature (Harpaz and Chothia, 1994).

Figure S5.

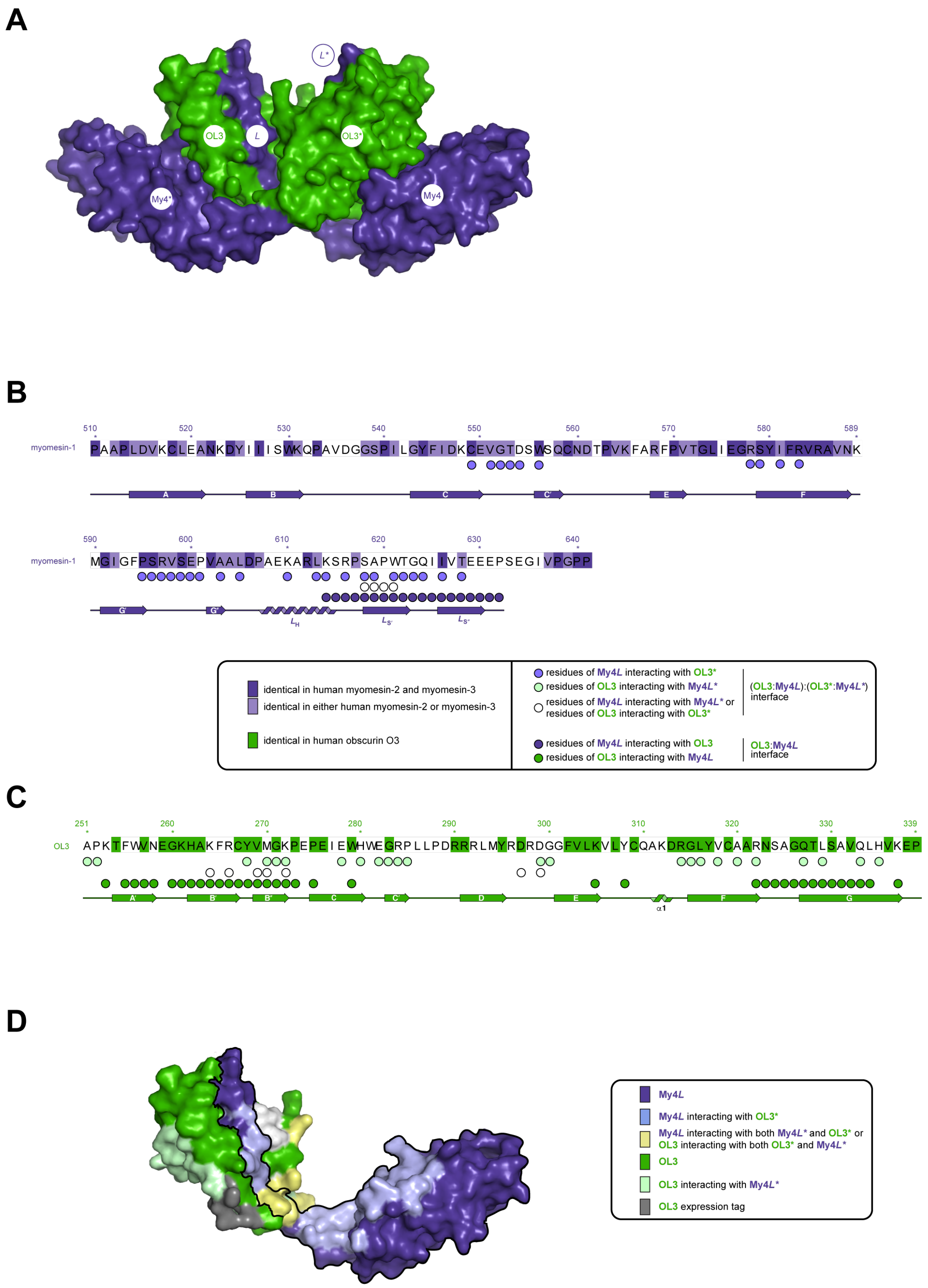
A
L*
OL3
L
OL3*
My4*
My4
B
myomesin-1
510
520
530
540
550
560
570
580
589
590
600
610
620
630
640
identical in human myomesin-2 and myomesin-3
identical in either human myomesin-2 or myomesin-3
identical in human obscurin O3
residues of My4L interacting with OL3*
residues of OL3 interacting with My4L*
residues of My4L interacting with My4L* or residues of OL3 interacting with OL3*
(OL3:My4L):(OL3*:My4L*) interface
residues of My4L interacting with OL3
residues of OL3 interacting with My4L
OL3:My4L interface
C
OL3
251
260
270
280
290
300
310
320
330
339
D
My4L
My4L interacting with OL3*
My4L interacting with both My4L* and OL3* or OL3 interacting with both OL3* and My4L*
OL3
OL3 interacting with My4L*
OL3 expression tag

Figure S5 (related to Figure 2A). (OL3:My4*L*)$_2$ homodimer. (A) Surface representation of the crystallographic (OL3:My4*L*)$_2$ homodimer. My4*L* and OL3 are in slate blue and green, respectively. An asterisk is used to identify domains belonging to the second OL3:My4*L* complex; (B-C) Sequence and secondary structure of My4*L* (B) and OL3 (C). Amino acids are color-coded according to sequence identity as shown by the conservation bar. Colored circles identify residues of one domain contacting the other as indicated in the inset; (D) Homodimerization contact map. Light blue, light green, and yellow patches on a single OL3:My4*L* complex highlight residues interacting with My4*L**, OL3*, or both domains, respectively. The outline of My4*L* is shown as a thick black line for clarity.

Figure S6.

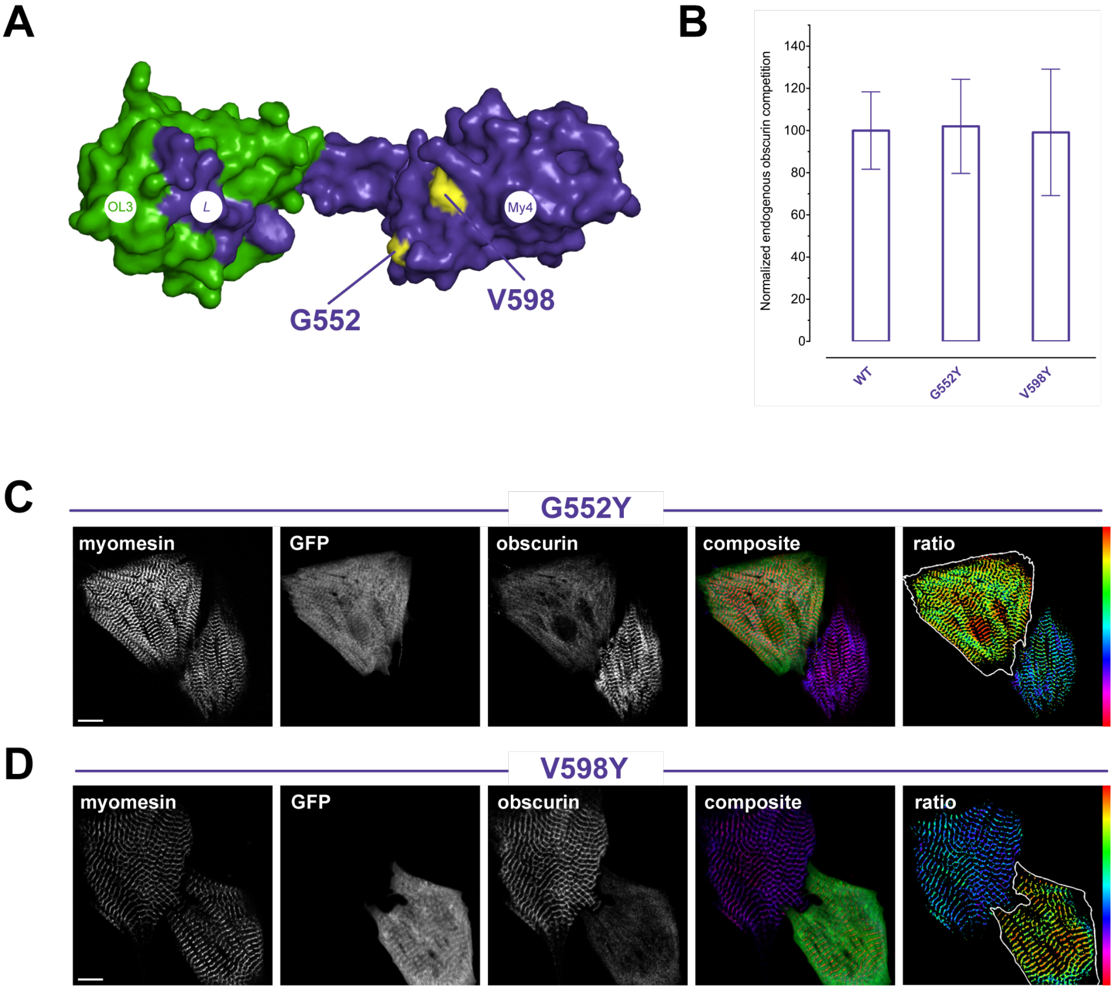
A
OL3
L
My4
G552
V598
B
Normalized endogenous obscurin competition
140
120
100
80
60
40
20
0
WT
G552Y
V598Y
C
G552Y
myomesin
GFP
obscurin
composite
ratio
D
V598Y
myomesin
GFP
obscurin
composite
ratio

Figure S6 (related to Figure 4). Controls for the competition assay in NRCs. (A) Surface representation of the OL3:My4*L* complex. Residues G552 and V598 that are not involved in establishing the OL3:My4*L* interface are highlighted in yellow. (B) Quantification of endogenous obscurin displaced in NRCs expressing GFP-fused wild-type My4*L*My5 (n=11) and its G552Y (n=10) and V598Y (n=10) variants. As expected, these variants compete endogenous obscurin like the wild-type counterpart as their amino acid replacement does not interfere with OL3 binding; (C-D) Example of the competitive effect of overexpressed GFP-fused My4*L*My5 G552Y (C) and V598Y (D). The separate channels for endogenous myomesin, GFP, endogenous obscurin, as well as the combined and ratiometric images with overlaid GFP mask for the outline of the transfected cell are shown. The false-color scale range indicator shows an increased obscurin/myomesin ratio. The scale-bar is 10 μm.

Figure S7.

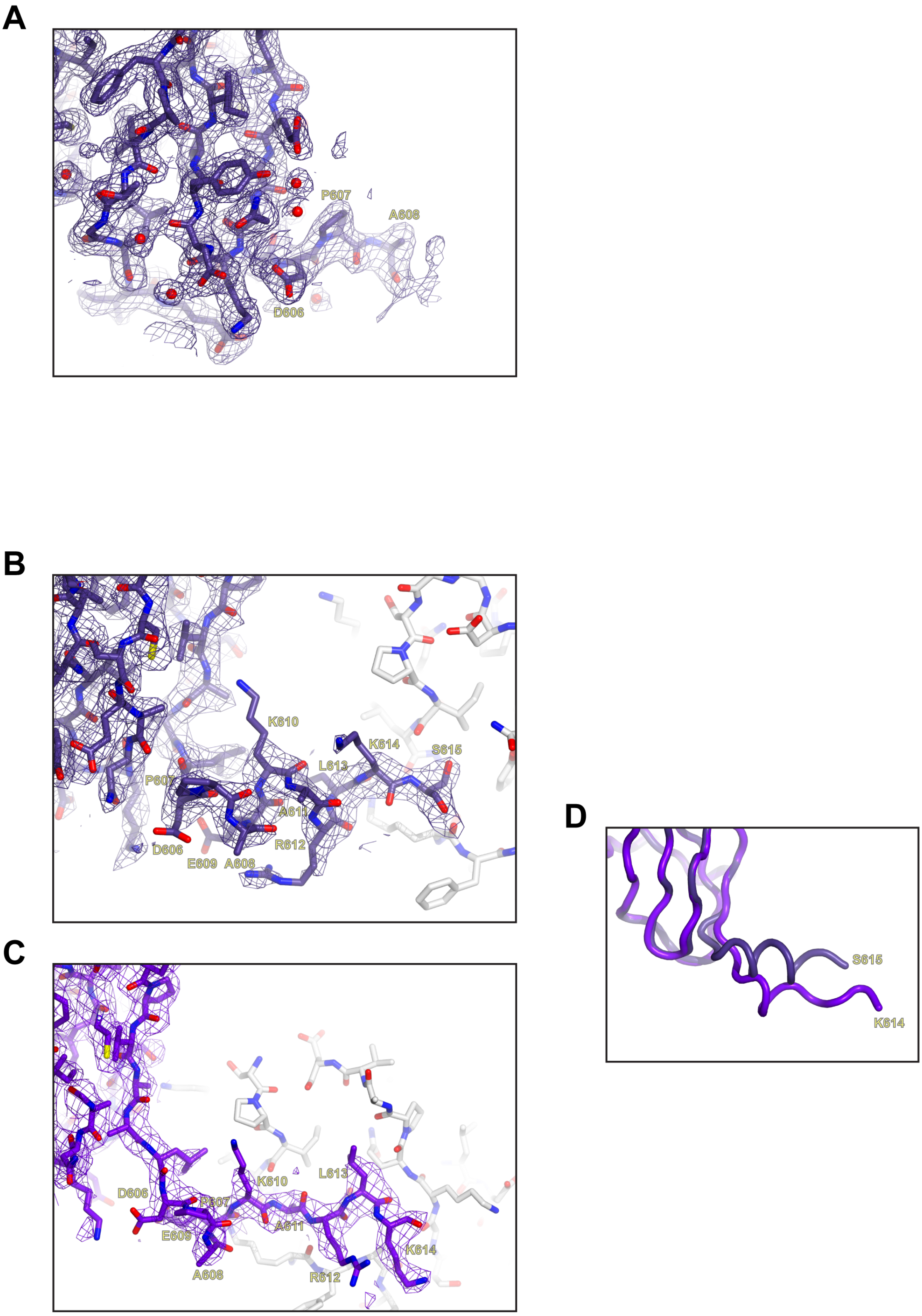
A
P607
A608
D606
B
K610
K614
S615
L613
P607
A611
D606
R612
E609
A608
C
L613
K610
D606
P607
A611
E609
K614
A608
R612
D
S615
K614

Figure S7 (related to Figure 5). The C-terminal helix is generally disordered in My4L_H (myomesin residues 510-618) crystals. (A-C) Close-up on the C-terminal region of My4L_H in space group $P6_5$ (2.05 Å resolution) (A) and in space group $P2_1$ (2.80 Å resolution) (B-C). $2mF_o$-DF_c electron density maps are shown in blue at the 1.0σ contour level. My4L_H models are shown as sticks in slate blue. Symmetry related molecules are in grey. Oxygen, nitrogen, sulphur atoms are in red, blue, yellow, respectively. In space group $P6_5$ all four My4L_H molecules in the a.u. display clear electron density until residue A608 while residues D609-S618 cannot be modelled (A). In space group $P2_1$ (six molecules/a.u) the L_H region folds into a helix in one molecule (B) whilst in another molecule this region is more extended (C). All other molecules display a disordered C-terminus that could not be modelled; (D) Cartoon tube superposition of My4L_H C-termini in helical and extended conformations.

Figure S8.

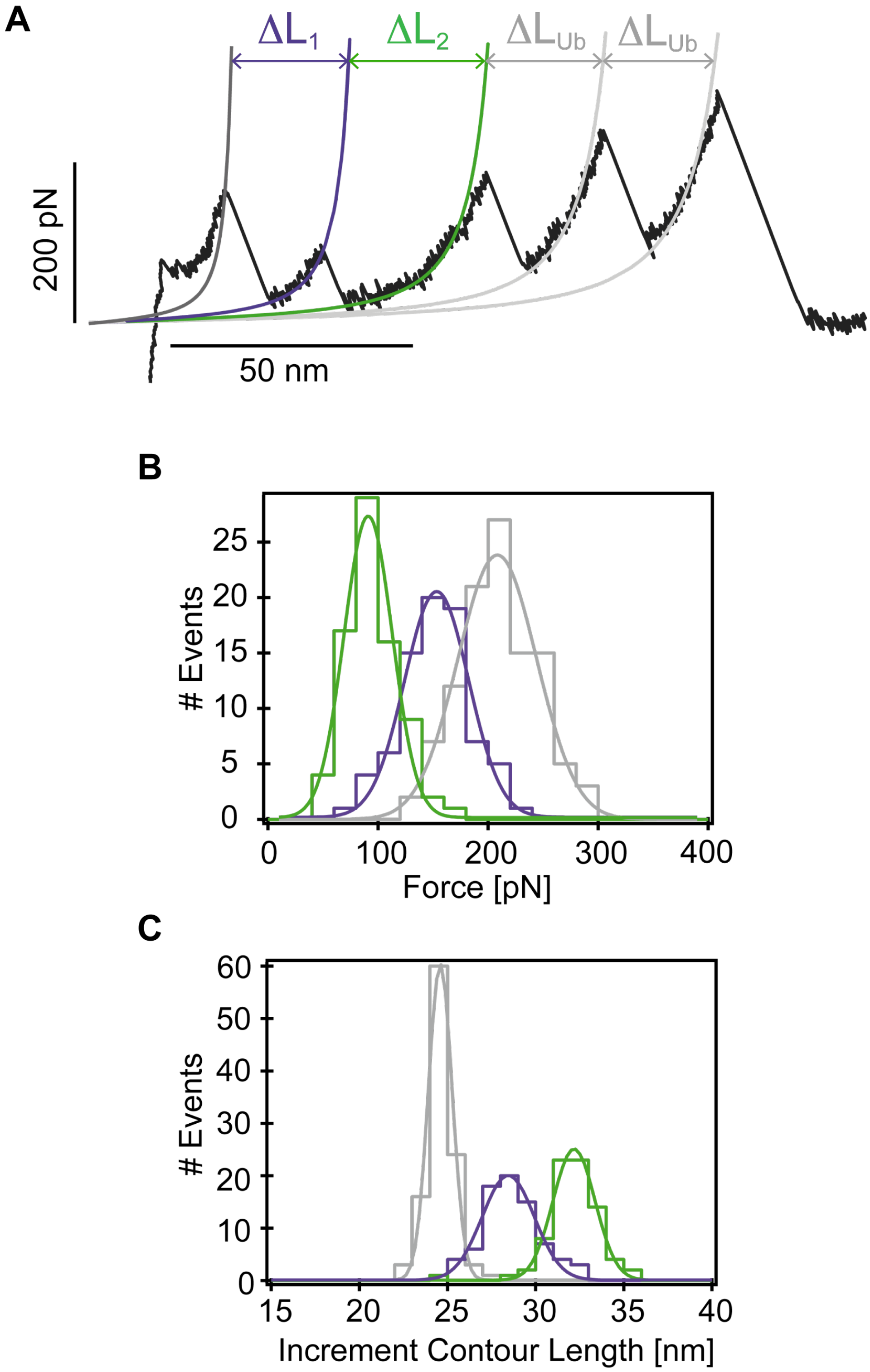

A
ΔL1
ΔL2
ΔLUb
ΔLUb
200 pN
50 nm
B
Events
25
20
15
10
5
0
0
100
200
300
400
Force [pN]
C
Events
60
50
40
30
20
10
0
15
20
25
30
35
40
Increment Contour Length [nm]

Figure S8 (related to Figure 6). Force spectroscopy measurements on the ubiquitin-*L*-(connector)$_{68}$-OL3-ubiquitin polyprotein. (A) Typical force-extension trace for the ubiquitin-*L*-(connector)$_{68}$-OL3-ubiquitin polyprotein. Fit to the worm-like chain model (WLC) provides contour length increases, ΔL, from single domains unfolding events; (B) Histogram of domain unfolding forces. Force peaks are estimated at 143±29 pN (n=78), 81±22 pN (n=78), and 198±35 (n=107), for *L* unlatching, OL3 and ubiquitin unfolding, respectively; (C) Histogram of measured contour length increases. ΔL_1=27.9±1.5 nm (n=78), ΔL_2=27.9±1.5 nm (n=78), and ΔL_{Ub}=23.8±0.6 nm (n=107), for *L* unlatching, OL3 and ubiquitin unfolding, respectively. In (B) and (C) colored curves are Gaussian fits.

Legend Movie S1 (related to Figure 5). Conformational transition from the compact conformation (as observed by SAXS) to the more extended geometry (as observed in the crystallographic dimer) for the OL3-My4*L* complex. My4*L* and OL3 are shown as surface representation in slate blue and green, respectively.

References

Bricogne, G., Blanc, E., Brandl, M., Flensburg, C., Keller, P., Paciorek, W., Roversi, P., Sharff, A., Smart, O.S., Vonrhein, C.*, et al.* (2011). BUSTER version 2.10.0. Global Phasing Ltd., Cambridge, United Kingdom.

Emsley, P., Lohkamp, B., Scott, W.G., and Cowtan, K. (2010). Features and development of Coot. Acta Crystallogr. D, Biol. Crystallogr. *66*, 486-501.

Franke, D., and Svergun, D.I. (2009). DAMMIF, a program for rapid ab-initio shape determination in small-angle scattering. J. Appl. Crystallogr. *42*, 342-346.

Harpaz, Y., and Chothia, C. (1994). Many of the immunoglobulin superfamily domains in cell adhesion molecules and surface receptors belong to a new structural set which is close to that containing variable domains. J. Mol. Biol. *238*, 528-539.

Matthews, B.W. (1968). Solvent content of protein crystals. J. Mol. Biol. *33*, 491-497.

Mrosek, M., Labeit, D., Witt, S., Heerklotz, H., von Castelmur, E., Labeit, S., and Mayans, O. (2007). Molecular determinants for the recruitment of the ubiquitin-ligase MuRF-1 onto M-line titin. FASEB J. *21*, 1383-1392.

Murshudov, G.N., Skubak, P., Lebedev, A.A., Pannu, N.S., Steiner, R.A., Nicholls, R.A., Winn, M.D., Long, F., and Vagin, A.A. (2011). REFMAC5 for the refinement of macromolecular crystal structures. Acta Crystallogr. D, Biol. Crystallogr. *67*, 355-367.

Pernigo, S., Fukuzawa, A., Bertz, M., Holt, M., Rief, M., Steiner, R.A., and Gautel, M. (2010). Structural insight into M-band assembly and mechanics from the titin-obscurin-like-1 complex. Proc. Natl. Acad. Sci. USA *107*, 2908-2913.

Pernigo, S., Fukuzawa, A., Pandini, A., Holt, M., Kleinjung, J., Gautel, M., and Steiner, R.A. (2015). The crystal structure of the human titin:obscurin complex reveals a conserved yet specific muscle M-band zipper module. J. Mol. Biol. *427*, 718-736.

Petoukhov, M.V., Franke, D., Shkumatov, A.V., Tria, G., Kikhney, A.G., Gajda, M., Gorba, C., Mertens, H.D., Konarev, P.V., and Svergun, D.I. (2012). New developments in the program package for small-angle scattering data analysis. J Appl. Crystallogr. *45*, 342-350.

Popa, I., Kosuri, P., Alegre-Cebollada, J., Garcia-Manyes, S., and Fernandez, J.M. (2013). Force dependency of biochemical reactions measured by single-molecule force-clamp spectroscopy. Nat. Protoc. *8*, 1261-1276.

Schlierf, M., Li, H., and Fernandez, J.M. (2004). The unfolding kinetics of ubiquitin captured with single-molecule force-clamp techniques. Proc. Natl. Acad. Sci. USA *101*, 7299-7304.

Seeliger, D., Haas, J., and de Groot, B.L. (2007). Geometry-based sampling of conformational transitions in proteins. Structure *15*, 1482-1492.

Svergun, D.I. (1999). Restoring low resolution structure of biological macromolecules from solution scattering using simulated annealing. Biophys. J. *76*, 2879-2886.

Vagin, A., and Teplyakov, A. (2010). Molecular replacement with MOLREP. Acta Crystallogr. D, Biol. Crystallogr. *66*, 22-25.

Volkov, V.V., and Svergun, D.I. (2003). Uniqueness of ab initio shape determination in small-angle scattering. J. Appl. Crystallogr. *36*, 860-864.
